# Binding Differences of the Peptide‐Substrate–Binding Domain of Collagen Prolyl 4‐Hydroxylases I and II for Proline‐ and Hydroxyproline‐Rich Peptides

**DOI:** 10.1002/prot.26839

**Published:** 2025-05-19

**Authors:** M. Mubinur Rahman, Ramita Sulu, Bukunmi Adediran, Hongmin Tu, Antti M. Salo, Sudarshan Murthy, Johanna Myllyharju, Rik K. Wierenga, M. Kristian Koski

**Affiliations:** ^1^ Faculty of Biochemistry and Molecular Medicine University of Oulu Oulu Finland; ^2^ Biocenter Oulu, University of Oulu Oulu Finland

**Keywords:** collagen, hydroxyproline, PPII, proline, prolyl 4‐hydroxylase, PSB domain

## Abstract

Collagen prolyl 4‐hydroxylase (C‐P4H) catalyzes the 4‐hydroxylation of Y‐prolines of the XYG‐repeat of procollagen. C‐P4Hs are tetrameric α_2_β_2_ enzymes. The α‐subunit provides the N‐terminal dimerization domain, the middle peptide‐substrate–binding (PSB) domain, and the C‐terminal catalytic (CAT) domain. There are three isoforms of the α‐subunit, complexed with a β‐subunit that is protein disulfide isomerase, forming C‐P4H I‐III. The PSB domain of the α‐subunit binds proline‐rich peptides, but its function with respect to the prolyl hydroxylation mechanism is unknown. An extended mode of binding of proline‐rich peptides (PPII, polyproline type‐II, conformation) to the PSB‐I domain has previously been reported for the PPG‐PPG‐PPG and P9 peptides. Crystal structures now show that peptides with the motif **PxGP** (PPG‐**PRG**‐**P**PG, PPG‐**PAG**‐**P**PG) (where x, at Y‐position 5, is not a proline) bind to the PSB‐I domain differently, more deeply, in the peptide‐binding groove. The latter mode of binding has previously been reported for structures of the PSB‐II domain complexed with these PxGP‐peptides. In addition, it is shown here by crystallographic binding studies that the POG‐PAG‐POG peptide (with 4‐hydroxyprolines at Y‐positions 2 and 8) also adopts the PxGP mode of binding to PSB‐I as well as to PSB‐II. Calorimetric binding studies show that the affinities of these peptides are lower for PSB‐I than for PSB‐II, with, respectively, *K*_*D*_ values of about 70 μM for PSB‐I and 20 μM for PSB‐II. The importance of these results for understanding the reaction mechanism of C‐P4H, in particular concerning the function of the PSB domain, is discussed.

**Abbreviations**
Bis‐Tris: 1,3‐bis[tris(hydroxymethyl)methylamino]propane
CAT‐domain: the catalytic domain of the α‐subunit
CD: circular dichroism
CHES: 2‐(cyclohexylamino)ethanesulfonic acid
C‐P4H: collagen prolyl 4‐hydroxylase
CRTAP: cartilage associated protein
DD‐I: double domain construct of the α‐subunit of C‐P4H‐I, consisting of the N‐terminal dimerization domain and the middle PSB domain
ESRF: European Synchrotron Radiation facility
HPLC: high‐performance liquid chromatography
ITC: isothermal titration calorimetry
MALS: multi‐angle light scattering
MES: 2‐(N‐morpholino)ethanesulfonic acid
MOPS: 3‐(N‐morpholino)propanesulfonic acid
MPD: 2‐methyl‐2,4‐pentanediol
O: one‐letter code for the (2*S*,4*R*)‐4‐hydroxyproline amino acid (3‐letter code: Hyp)
P3H1: prolyl 3‐hydroxylase 1
P4H: prolyl 4‐hydroxylase
P9: (Pro)_9_ peptide
PDB: Protein Data Bank
PDI: protein disulfide isomerase
(PPG)_3_
: PPG‐PPG‐PPG peptide
PPII conformation: the polyproline type‐II conformation
PSB: peptide‐substrate–binding domain of C‐P4H
PxGP‐peptides: PPG‐PAG‐PPG, PPG‐PEG‐PPG, PPG‐PRG‐PPG, and POG‐PAG‐POG
RI detector: refractive index detector
SEC: size‐exclusion‐chromatography
TPR: tetratricopeptide repeat
Tris: tris(hydroxymethyl)aminomethane

## 1 Introduction

**FIGURE 1:**
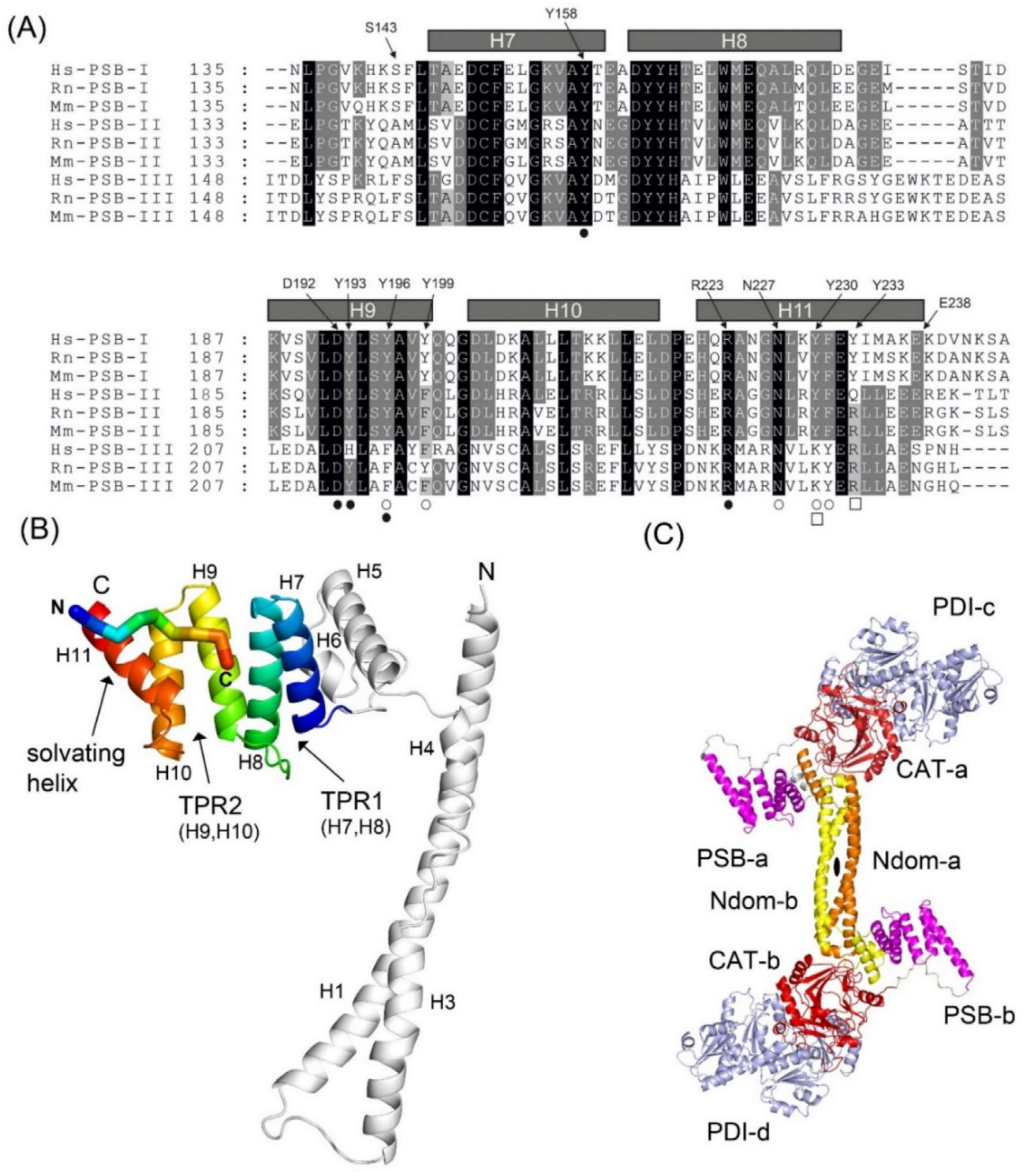
Sequence and structure information of the PSB domain, the DD construct and the α_2_β_2_ C‐P4H tetramer. (A) Multiple sequence alignment of PSB‐I, PSB‐II and PSB‐III amino acid sequences. Hs, Rn, Mn refer to the human, rat and mouse sequences. Fully conserved residues are shown as white letters on black background. Similar residues are shown as white letters on gray background. The sequence numbering above the sequences refers to the residue numbering scheme of PSB‐I, as used previously, which is the numbering of the mature α‐I subunit, after cleavage of the signal peptide [8]. The open squares, the open circles and the black dots below the sequences identify the residues which define the proline binding P1‐, P5‐, and P8‐pockets, respectively. (B) Crystal structure of one chain of the dimeric C‐P4H‐I double domain (DD‐I) construct (PDB ID 4BTB), consisting of the N‐terminal dimerization domain (gray) and the PSB domain of the α‐I subunit. The latter domain as well as its bound peptide, P9, are in rainbow coloring from blue (at the N‐terminus) to red (at the C‐terminus). The helices H7, H8, H9, H10, H11, which form the PSB domain are labeled. H7/H8 and H9/H10 form two TPR units and H11 is known as the solvating helix. The helices H1, H3, H4, H5, and H6 of the N‐terminal dimerization domain are also labeled. (C) AlphaFold model of the C‐P4H‐II α_2_β_2_ tetramer [9]. The two α‐II subunits are colored orange (Ndom‐a), magenta (PSB‐a), red (CAT‐a) and yellow (Ndom‐b), magenta (PSB, b), red (CAT‐b), respectively. The two β/PDI subunits (PDI‐c, PDI‐d) are colored light‐blue.

Collagen prolyl 4‐hydroxylases (C‐P4Hs), located in the lumen of the endoplasmic reticulum, catalyze the hydroxylation of peptidyl prolines to 4‐hydroxyprolines at the Y‐position of the XYG repeats of procollagen chains [1]. The most frequently observed residues at these X and Y positions are prolines [2]. Three collagen chains assemble into the collagen triple helix, which requires a glycine at every third position of this repeat. Hydroxylation of Y‐prolines is critically important for the stability of the collagen triple helix [2]. These collagens have important functions in the extracellular matrix and consequently play a role in several pathological states, like fibrosis and cancer [3, 4]. C‐P4H is an α_2_β_2_ heterotetramer with three isoenzymes in mammals, each with a unique α‐subunit, referred to as α‐I, α‐II, and α‐III [1]. C‐P4H‐I and C‐P4H‐II are the most extensively characterized C‐P4Hs, and their β‐subunit is identical to PDI, as also proposed for C‐P4H‐III. Recent studies have shown that C‐P4H‐I and C‐P4H‐II have sequence specificity toward different—XPG—collagen sites [5, 6]. The amino acid sequence identity between human α‐I and α‐II is 65%, and that of α‐III with α‐I and α‐II is 35% and 37%, respectively [7]. The α‐subunit is composed of three domains: the N‐terminal dimerization domain, the middle peptide‐substrate–binding (PSB) domain, consisting of about 100 residues (Figure 1A), and the C‐terminal catalytic (CAT) domain. The CAT domain adopts the double stranded beta helix (DSBH) fold, like other Fe(II) and 2‐oxoglutarate dependent oxygenases [10], that use molecular oxygen in the hydroxylation reaction [1, 11].

**FIGURE 2:**
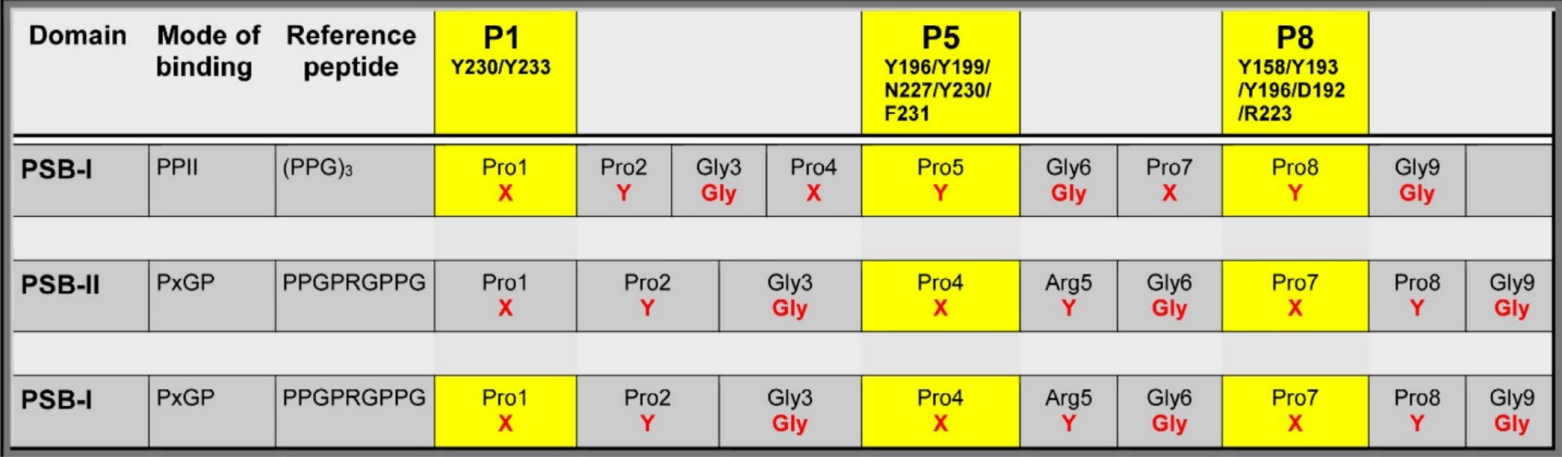
Schematic overview identifying the prolines of the PSB bound reference peptide, that interact with the respective proline binding pockets (P1, P5, and P8) of the PSB domain of PSB‐I (PPII mode of binding) [12], of PSB‐II (PxGP mode of binding) [17] and of PSB‐I (PxGP mode of binding) (these studies). These pockets are shaped by the side chains of the listed residues, using the human PSB‐I sequence numbering (Figure 1A). The proline residues of the XYG‐repeats of the bound peptide that are highlighted in yellow, bind in the given pocket of the PSB domain. A functional P1‐pocket does not exist in PSB‐II, as Tyr233 is not a conserved residue, being a glutamine in human PSB‐II. This scheme also visualizes that in the PPII mode of binding Y‐prolines bind in the P5‐ and P8‐pockets, whereas in the PxGP mode of binding X‐prolines bind in these pockets.

Crystal structures have been reported of a dimer of the double domain construct of human C‐P4H‐I (DD‐I), which consists of its N‐terminal dimerization domain and its PSB domain [12] (Figure 1B), showing the coiled‐coil mode of interactions between the dimerization domains of the α_2_ dimer of the α_2_β_2_ complex. A crystal structure has also been reported for a complex of the CAT domain of the α subunit of human C‐P4H‐II, complexed with the β/PDI subunit [9]. The protein crystallographic studies of these two truncated versions of the α_2_β_2_ tetramer (the DD‐I homodimer and the CAT‐PDI‐II heterodimer) have provided a model of the α_2_β_2_ tetramer using the AlphaFold structure prediction methods [13] (Figure 1C), showing the position and orientation of the PSB domain with respect to the CAT domain [9]. In this model of the α_2_β_2_ tetramer, the PSB domain is protruding out of the core of the α_2_β_2_ complex. The PSB‐I domain of C‐P4H‐I was originally identified based on its property of tightly binding proline‐rich peptide substrates and inhibitors [14] and its crystal structure has been described [15]. It has the tetratricopeptide repeat (TPR) fold [16] consisting of 5 helices known as helices H7, H8, H9, H10, and H11, in which H7/H8 and H9/H10 form two TPR units, and H11 is referred to as the solvating helix (Figure 1B). The peptide‐binding groove is mainly formed by the parallel helices H7, H9, and H11, as shown by subsequent crystallographic binding studies of the DD‐I construct with the proline‐rich substrate, (PPG)_3_, and inhibitor, P9 [12] to its PSB domain. The directionality of the mode of binding of these peptides is the same as observed in peptide complexes of other TPR domains [12, 15]. Several of the proline side chains of these peptides interact with conserved tyrosine side chains in three different proline binding pockets of the PSB domain (Figures 1A and 2). Interactions of proline and tyrosine side chains are very often observed in structures of proteins complexed with proline‐rich peptides [18, 19].

The role of the PSB domain in the hydroxylation mechanism of C‐P4H is not known, but the binding affinities of several (PPG)_n_ peptide substrates and poly(L‐Pro) inhibitors to the PSB‐I and PSB‐II domains are very similar to the *K*_*M*_ and *K*_i_ values of C‐P4H‐I and C‐P4H‐II for these peptides, and these affinities are significantly higher for C‐P4H‐I as compared with C‐P4H‐II [15, 20]. Furthermore, mutation of the conserved tyrosines Tyr193, Tyr196, and Tyr230, located in the peptide‐binding groove of the PSB domain of C‐P4H‐I, to alanines significantly increases the *K*_*M*_ value of the substrate peptide (PPG)_10_ and the IC50 value of a poly(L‐Pro) inhibitor [15]. These data strongly suggest a functional importance of the PSB domain for the hydroxylation catalysis by C‐P4H. Protein crystallographic studies of complexes with peptides have also been done with the PSB construct of human C‐P4H‐II [17] as well as with a close homolog of the CAT domain (the monomeric algal P4H) [21]. Superimposing the structures of these PSB‐ and CAT‐peptide complexes on the PSB and CAT domains of the α_2_β_2_ tetramer model predicts that the C‐terminus of the PSB‐bound peptide points to the N‐terminus of the peptide bound at the active site of the CAT domain [9].

The DD‐I peptide binding studies with P9 and (PPG)_3_ show that these peptides bind in the extended polyproline type‐II (PPII) conformation [2, 22] to the PSB domain [12]. These structures have identified three proline binding pockets, referred to here as the P1‐pocket (near Tyr230 and Tyr233), the P5‐pocket (shaped by Tyr196, Tyr199, Asn227, Tyr230 and Phe231) and the P8‐pocket (shaped by Tyr158, Tyr193, Tyr196 as well as by the Asp192–Arg223 salt bridge), as schematically visualized in Figure 2. Calorimetric binding studies with this DD‐I construct showed that (PPG)_3_ and P9 have high affinity for this construct. More recent peptide binding studies of the PSB‐II construct of C‐P4H‐II [17] showed that the latter two peptides have low affinity for PSB‐II, whereas the peptides PPG‐PAG‐PPG, PPG‐PEG‐PPG, and PPG‐PRG‐PPG have high affinity. Crystallographic binding studies of this PSB‐II construct with these peptides show that these peptides bind to the PSB domain in a conformation that is different from the extended PPII conformation observed in the structures of the (PPG)_3_ and P9 complexes of the PSB‐I domain of DD‐I. Nevertheless, the P5‐ and P8‐pockets are used for binding a proline side chain in both complexes [17] (Figure 2). The binding motif of these PPG‐P(A/R/E)G‐PPG peptides to the P5‐ and P8‐pockets of PSB‐II has been referred to as the PxGP motif, in which the x‐residue corresponds to the Y‐residue of the XYG‐repeat. In the PxGP conformation, the residue x (at Y‐position 5 (Ala, Arg, or Glu) of the PPG‐PxG‐PPG peptide) and the glycine (at position 6 of the PPG‐PxG‐PPG peptide) of the PxGP motif adopt different conformations as compared with the extended PPII conformation of (PPG)_3_ and P9 bound to the PSB‐I domain. The conformation of this PxGP motif is such that residue x cannot be a proline [17]. The “deep” PxGP mode of binding allows for the main chain N and O atoms of residue x (at Y‐position 5) to form direct hydrogen bonds to the side chain oxygen (OD1) and nitrogen (ND2) atoms, respectively, of the highly conserved Asn227 [17], instead of by water‐mediated hydrogen bonds, as seen in the PPII mode of binding of (PPG)_3_ to PSB‐I [12].

Here the structures of the complexes of the PxGP‐peptides PPG‐PRG‐PPG and PPG‐PAG‐PPG with the PSB‐I construct are reported, showing that these peptides bind in the same conformation as seen in the corresponding PSB‐II complexes. It is also shown here by calorimetric binding assays that these PxGP‐peptides bind with good affinity to the PSB‐I domain. In addition, calorimetric and crystallographic binding studies of the PSB‐I construct and the PSB‐II construct with the POG‐PAG‐POG peptide (having a 4‐hydroxyproline residue, at the Y‐position 2 and 8) are also reported. The importance of the two modes of binding of proline‐rich peptides to the PSB domain is discussed in the context of the possible role of the PSB domain with respect to the substrate specificity and the reaction mechanism of C‐P4H.

## 2 Materials and Methods

### 2.1 Cloning, Expression, and Purification of Human C‐P4H PSB Constructs

The PSB‐I^143–238^ construct was cloned using standard molecular biology protocols, and a codon‐optimized *P4ha1* cDNA for the full‐length C‐P4H‐I α‐subunit (GenScript Biotech Corporation, New Jersey, USA) was used as the template. This construct was amplified using a forward primer containing an *Nde*I site (underlined) (5′‐TTTTTTCATATGAGCTTTCTGACC‐3′) and a reverse primer containing an *Xho*I site (underlined) (5′‐AAACTCGAGTTCTTTCGCCAT‐3′). The amplified product was digested with *Nde*I and *XhoI* and ligated into a pET22b(+) plasmid (Novagen, Wisconsin, USA) in‐frame with a C‐terminal His_6_‐tag, and its sequence (Ser143‐Glu238 (Figure 1A), with C‐terminal Leu‐Glu‐His_6_–tag) was verified by DNA sequencing in the Biocenter Oulu Sequencing Centre. PSB‐I^143–238^ was expressed using Rosetta(DE3)pLysS cells in LB medium containing 100 μg/mL ampicillin and 34 μg/mL chloramphenicol. The cells were grown at 37°C using a shaker at 200 rpm until the OD_600_ reached 0.6–0.8, after which protein expression was induced using 1‐mM isopropyl β‐D‐1‐thiogalactopyranoside and incubated at 30°C for another 4 h. The cells were harvested and resuspended in 20 mL of 20‐mM Bis‐Tris, 100 mM glycine, pH 6.8 lysis buffer. After cell lysis by sonication, the supernatant was loaded onto a 20 mL Ni‐NTA chelating Sepharose column, pre‐equilibrated with the lysis buffer. Unbound proteins were washed away with 100 mL of the same buffer, and the bound proteins were eluted with a 100 mL linear imidazole gradient (0.0–0.5 M). The eluted fractions were analyzed by SDS‐PAGE to identify PSB‐I containing fractions, which were pooled, concentrated using the Amicon ultracentrifugal filter (30 kDa molecular weight cut‐off) and loaded on a Superdex75 16/600 preparative grade size‐exclusion chromatography (SEC) column (GE Healthcare, Chicago, USA), pre‐equilibrated with a 20 mM Bis‐Tris, 100 mM glycine, pH 6.8, SEC buffer. Fractions with PSB‐I^143–238^ of high purity, based on SDS‐PAGE analysis, were pooled and concentrated to about 22 mg/mL (measured with a NanoDrop 1000, Thermo Scientific, Waltham, USA) and stored in the SEC buffer in 100 μL aliquots at −70°C. All characterizations, crystallization, and crystal testing experiments described below were performed in the Biocenter Oulu core facilities.

Additional experiments were done with a longer PSB‐I construct, PSB‐I^144–244^ (residues Phe144‐Ser244 (Figure 1A), extended by a C‐terminal Leu‐Glu‐(His)_6_–tag) and with the PSB‐II^142–236^ construct (residues Met142‐Glu236 (Figure 1A), preceded at the N‐terminus by a Met‐His_6_–tag). The expression and purification protocols for the PSB‐I^144–244^ construct [15] and the PSB‐II^142–236^ construct [17] have been described earlier.

### 2.2 Proline‐Rich Peptides

The peptides used in these studies were purchased from TAG Copenhagen A/S (Frederiksberg, Denmark). These peptides (9 residues) start with a free amino group and end with a free carboxylate group. The correct mass and the purity of the peptides (> 95% pure) were analyzed by the company using HPLC and mass spectrometry.

### 2.3 Multi‐Angle Light Scattering (MALS)

The MALS analysis was done with the PSB‐I^143–238^ construct. The used MALS instrument, MiniDAWN (Wyatt Technology, California, USA), was connected to a Shimadzu High Performance Liquid Chromatography (HPLC) system (Shimadzu, Kyoyo, Japan) including LC‐10Ai solvent delivery modules, SIL‐20AC autoSampler, and SPD‐M20A diode array detector. This system also included the Optilab refractive index (RI) detector (Wyatt Technology). A Superdex 200 Increase 10/300 GL (bed volume 24 mL, GE Healthcare) column was used for the SEC step. The column and the HPLC detectors were placed in a cooling cabinet with temperature ranging between +5°C and +7°C. The MALS and RI detectors were placed outside of the cabinet at room temperature (+20°C). The MALS analysis was done with the ASTRA V 7.3.2 software (Wyatt Technology).

### 2.4 Circular Dichroism (CD)

The CD spectrum and CD‐melting curve of purified PSB‐I^143–238^ were obtained using a Chirascan CD spectrophotometer (Applied Photophysics Ltd., Leatherhead, Surrey, UK) in a quartz cuvette with a 1‐mm path length. The protein sample was diluted to 0.08 mg/mL using milli‐Q water, the final buffer conditions being 0.1 mM Bis‐Tris, 0.5 mM glycine, pH 6.8. A wavelength scan from 185 to 260 nm (at 20°C) was used to analyze the secondary structure of the protein. Thermal denaturation was recorded by measuring the CD spectrum between 190 and 260 nm using a Quantum temperature controller with a 2°C step size at 1°C/min ramp rate with ±0.2°C tolerance, from 20°C to 90°C. Data analysis was carried out using Pro‐Data Viewer (Applied Photophysics Ltd.), CDNN (courtesy of and written by Dr. Gerald Böhm, 1997, Institut für Biotechnologie, Martin–Luther Universität Halle–Wittenberg, Germany, distributed by Applied Photophysics), and Global3 (Applied Photophysics Ltd.).

### 2.5 Isothermal Titration Calorimetry (ITC)

The protein samples of the PSB‐I^143–238^ and PSB‐II constructs were prepared at a concentration of 50 μM in SEC buffer, being 20 mM Bis‐Tris, 100 mM glycine, pH 6.8 for PSB‐I^143–238^ and 20 mM Tris, 50 mM NaCl, 50 mM glycine, pH 8.0 for PSB‐II (as described earlier [17]). The peptides were dissolved in SEC buffer at a concentration of 3 mM by diluting a 100 mM stock solution in water with SEC buffer. The ITC measurements were carried out at 25°C, using the MicroCal iTC‐200 (Malvern Panalytical Ltd., Malvern, UK). The peptide solution was titrated into a sample cell of 200 μL volume from a syringe that was rotating at 750 rpm. First, 0.4 μL of the peptide solution was injected with a duration of 0.8 s; then, 12 consecutive injections were done, each with a volume of 3 μL, a duration of 6 s, and 180 s spacing time between each injection. For each peptide, at first only buffer in the sample cell (without protein) was titrated, and data were collected as control. Then the data for the PSB sample titrated with the peptide‐containing buffer were collected. The titration data were processed with Origin 7 (OriginLab, Massachusetts, USA) specifically designed for ITC data analysis. The control data that measured the residual heat effect from the peptide dilution were subtracted from the data of the peptide titration with PSB in the sample cell. The integrated data were analyzed using the one‐set‐of‐sites fitting model with a fixed stoichiometry (*n* = 1). The average values and standard deviations were obtained from three measurements.

### 2.6 Crystallization, Data Collection, and Structure Refinement

The PSB‐I^143–238^ construct was cocrystallized with the peptides PPG‐PAG‐PPG, PPG‐PRG‐PPG, and POG‐PAG‐POG, and the PSB‐II construct was cocrystallized with POG‐PAG‐POG by using a sitting‐drop method and IQ 96‐well plates (SPT Labtech, Melbourn, UK). The used peptide concentrations in the protein buffer (SEC buffer) are listed in Table 1. The mosquito LCP nanodispenser (SPT Labtech) was used to make the crystallization drops. The crystallization plates were incubated at 4°C, and the Formulatrix Rock Imager RI54 was used for imaging the crystallization drops, and the in‐house developed IceBear software was used to monitor the crystallization results [24]. The PSB‐I^143–238^ peptide complexes crystallized in very similar conditions containing around 30% 2‐methyl‐2,4‐pentanediol (MPD), 50 mM MgCl_2_, 50‐mM KCl, and 100 mM MOPS with pH ranging from 6.5 to 7.0 (Table 1). In addition, 3% D‐galactose was used as an additive in the PSB‐I(PPG‐PAG‐PPG) crystallization experiments. The condition that was previously used for the crystallization of the PSB‐II peptide complexes [17] did not work for the crystallization of the PSB‐II(POG‐PAG‐POG) complex. Subsequently, diffracting crystals of PSB‐II(POG‐PAG‐POG) were obtained in two new conditions: (i) 20% PEG400, 10% isopropanol, 100‐mM MOPS, pH 7.5 and (ii) 2‐M ammonium sulfate, 10% dioxane, 100 mM MES, pH 5.5. The latter crystal form was used for the structure determination of this complex. For data collection, the PSB‐I(PPG‐PAG‐PPG), PSB‐I(PPG‐PRG‐PPG), PSB‐I(POG‐PAG‐POG), and PSB‐II(POG‐PAG‐PPG) crystals were frozen in a cold room by transfer into liquid nitrogen. No cryoprotectant was used for the freezing of these crystals. The diffraction properties of these crystals were first tested with the in‐house Microstar X8 rotating anode X‐ray generator (Bruker, Karlsruhe, Germany). High‐resolution data sets were subsequently collected by using synchrotron radiation, either at PETRA III, beamline P14 (EMBL, Hamburg, Germany) or at the European Synchrotron Radiation facility (ESRF) Massif beamline ID30A‐1 (Grenoble, France) (Table 1). The synchrotron data sets of PSB‐I(POG‐PAG‐POG) and PSB‐II(POG‐PAG‐POG) (collected at PETRA III) were integrated with XDS [25] and then scaled and merged with AIMLESS [26]. The PSB‐I(PPG‐PAG‐PPG) and PSB‐I(PPG‐PRG‐PPG) data sets were collected at the ESRF and autoprocessed with the autoPROC data processing pipeline [27] and scaled and merged with AIMLESS [26]. The structures were solved using PHASER [28], as implemented in the Phenix package [29, 30] and using PDB entries 2V5F and 6EVL for the PSB‐I and PSB‐II molecular replacement calculations, respectively. The PSB‐I(PPG‐PRG‐PPG) and PSB‐I(POG‐PAG‐POG) crystals belonged to the space group P2_1_2_1_2 and contained four copies in the asymmetric unit, whereas the PSB‐I(PPG‐PAG‐PPG) complex crystallized in the tetragonal space group P4_2_2_1_2, with two copies in the asymmetric unit. The bound peptides, as well as other solvent molecules, were built in their corresponding density once the structure of the protein part had been well refined. Chain C of the P2_1_2_1_2 crystal form had no density for the peptide. In the final structures, each of the chains of these PSB‐I structures interacts with one MOPS molecule (present in the well solution buffer). Its binding site is at a crystal contact region, extending toward the peptide‐binding groove, and in the peptide‐liganded chains, it also interacts with the bound peptide. The PSB‐II(POG‐PAG‐POG) crystal form is the same as described previously for the PSB‐II peptide complexes, with one chain per asymmetric unit [17]. The structures were refined with the Phenix package, and COOT [31] was used for model building in the electron density maps. The last refinement cycles were done with REFMAC5 [32] in the CCP4 Cloud [33]. The final coordinates were validated with MolProbity [23] and the PDB validation report [34, 35]. A summary of the refinement statistics is presented in Table 1.

**TABLE 1:** Crystallization, data collection, and statistics of data processing and refinement.

Data set (peptide)	PSB‐I (PPG‐PAG‐PPG)	PSB‐I (PPG‐PRG‐PPG)	PSB‐I (POG‐PAG‐POG)	PSB‐I^144–244^ (no peptide)	PSB‐II (POG‐PAG‐POG)
PDB ID	9HT8	9HPQ	9HRE	2V5F	9HTD
Construct	S143‐E238	S143‐E238	S143‐E238	F144‐S244	M142‐E236
Protein buffer (SEC buffer)	20 mM Bis‐Tris, 100 mM glycine, pH 6.8	20 mM Bis‐Tris, 100 mM glycine, pH 6.8	20 mM Bis‐Tris, 100 mM glycine, pH 6.8	20 mM Bis‐Tris, 100 mM glycine, pH 6.8	20 mM Tris, 50 mM glycine, 50 mM NaCl, pH 8.0
Protein (mg/mL)	11	11	11	10	8
Crystallization conditions (well solution)	32% MPD, 50 mM MgCl_2_, 50 mM KCl, 100 mM MOPS, 3% D‐galactose, pH 6.5	30% MPD, 50 mM MgCl_2_, 50 mM KCl, 100 mM MOPS, pH 7.0	30% MPD, 48 mM MgCl_2_, 50 mM KCl, 100 mM MOPS, pH 7.0	1 M sodium citrate, 100 mM CHES, pH 9.5	2 M (NH_4_)_2_SO_4_, 10% dioxane, 100 mM MES, pH 5.5
Peptide (mM)^a^	5	2	5	—	2
X‐ray source	ESRF, MASSIF‐1	ESRF, MASSIF‐1	PETRA III, P14	Enraf‐Nonius, FR591, Bruker	PETRA III, P14
Wavelength (Å)	0.965	0.966	0.976	1.542	0.976
Data processing					
Unit cell parameters (Å, °)	*a* = 84.64, *b* = 84.64, *c* = 90.17, *α* = *β* = *γ* = 90.0	*a* = 82.42, *b* = 86.32, *c* = 91.13, *α* = *β* = *γ* = 90.0	*a* = 81.04, *b* = 85.56, *c* = 92.51, *α* = *β* = *γ* = 90.0	*a* = 37.47, *b* = 59.05, *c* = 61.67, *α* = *β* = *γ* =90.0	*a* = 56.65, *b* = 56.65, *c* = 68.83, *α* = *β* = 90.0, *γ* = 120.0
Space group	P4_2_2_1_2	P2_1_2_1_2	P2_1_2_1_2	P2_1_2_1_2_1_	P3_2_21
Resolution range (Å)^b^	90.17–2.15 (2.21–2.15)	49.88–2.15 (2.25–2.17)	92.51–2.05 (2.11–2.05)	42.65–2.03 (2.14–2.03)	49.06–1.75 (1.78–1.75)
*V*_*m*_ (Å^3^/Da)	3.1	2.7	3.1	2.6	2.5
Molecules per asymmetric unit	2	4	4	1	1
Number of reflections	201 517 (15944)	164 217 (15851)	267 861 (20752)	51 588 (4201)	83 956 (4905)
Redundancy	10.9 (10.9)	4.7 (4.7)	6.6 (6.8)	5.6 (3.4)	6.6 (6.8)
Completeness (%)	99.5 (96.6)	99.6 (99.6)	98.5 (97.2)	98.8 (93.2)	97.1 (98.4)
*I*/*s* (I)	19.8 (1.0)	13.5 (1.1)	14.8 (1.6)	21.0 (10.1)	14.2 (1.3)
*R*_merge_ (%)	5.3 (227.5)	5.8 (141.2)	7.2 (165.0)	5.0 (16.4)	6.3 (107.5)
*R*_pim_ (%)	1.7 (71.3)	2.9 (71.4)	3.0 (67.6)	2.0 (11.0)	2.6 (43.3)
CC (1/2)	0.999 (0.548)	0.999 (0.482)	0.999 (0.468)		0.998 (0.556)
Wilson *B*‐factor (Å^2^)^c^	63.3	50.6	40.6	27.1	27.8
Model refinement					
Resolution (Å)	59.92–2.15	49.88–2.17	62.89–2.05	42.65–2.03	49.06–1.75
*R*_work_ (%)	19.8	22.5	19.7	19.6	16.5
*R*_free_ (%)	24.1	25.0	21.8	26.5	19.5
Total number of reflections	18 369	34 846	40 403	8657	12 783
Number of non‐hydrogen atoms	1864	3754	3753	995	882
Protein	1660	3341	3353	799	762
Peptide (A/B/D)	34/48/—	40/54/40	27/55/40	60^d^	40
Other ligands	85	149	149	—	25
Waters	37	130	129	136	55
Rmsd bond length (Å)	0.007	0.007	0.008	0.013	0.011
Rmsd bond angle (°)	1.6	1.5	1.7	1.5	1.9
Average *B*‐factor					
All atoms	84.3	64.8	61.9	19.4	40.3
Protein (Å^2^)	82.5	63.8	59.9	16.5	38.2
Peptide (A/B/D) (Å^2^)	97.7/115.6/—	80.7/73.7/76.9	86.3/84.2/101.2	40.9^d^	48.4
Other ligands (Å^2^)	97.1	76.2	81.2	—	68.3
Water molecules (Å^2^)	81.5	65.5	65.2	27.3	49.8
Ramachandran plot^e^					
Favored (%)	98.1	100.0	99.8	97.0	99.0
Allowed (%)	1.9	0.0	0.2	2.0	1.0
Outliers (%)	0.0	0.0	0.0	1.0	0.0

^a^ Peptide concentration in the protein buffer.

^b^ The numbers in parentheses define the high resolution shell.

^c^ As given in the PDB validation report.

^d^ This peptide is the His_6_‐tag of a crystallographically related chain, which is bound in the peptide‐binding groove.

^e^ Calculated by MolProbity [23].

Crystals of the longer PSB‐I^144–244^ construct were obtained by using the hanging drop vapor diffusion crystallization method. The crystals grew at 4°C in the presence of 1‐M sodium citrate and 0.1‐M CHES, pH 9.5 (Table 1). Prior to data collection, the crystal was frozen in liquid nitrogen using paraffin oil as a cryoprotectant. The data were measured at 100 K at the home source, a FR591 rotating anode (Bruker, Karlsruhe, Germany) generator equipped with a MAR345 image plate (MAR, Hamburg, Germany). The data were processed and scaled, merged by the programs iMOSFLM [36] and SCALA [37], respectively. The structure was solved by molecular replacement by the program PHASER [28] using the previously determined PSB‐I structure (PDB ID 1TJC, [15]), after removing solvent molecules, as a model. There is one molecule per asymmetric unit. The structure was refined using REFMAC5. The program COOT [31] was used for model building. When inspecting the initial maps, significant residual density was observed in the peptide‐binding groove. This density could be modeled as a (His)_6_‐peptide, and it was noted that this peptide fragment could be the C‐terminal His_6_‐tag of a crystallographically related molecule, being connected to it by a disordered region of several residues for which no structure could be built in the electron density map. The final refinement statistics are listed in Table 1.

### 2.7 Structure Analysis

Chain B of the PPG‐PRG‐PPG crystallized complex of PSB‐I^143–238^ is used when referring to the PPG‐PRG‐PPG mode of binding. Similarly, chain B is used of the PPG‐PAG‐PPG crystallized complex of PSB‐I^143–238^ and of the POG‐PAG‐POG crystallized complex of PSB‐I^143–238^. For the structure of unliganded PSB‐I^143–238^, chain C of the PPG‐PRG‐PPG PSB‐I^143–238^ crystallized complex is used. The other structure of PSB‐I included in these structure comparisons is the structure of the longer PSB‐I^144–244^ variant, as obtained from a crystal grown without adding a proline‐rich peptide in the crystallization drop. The latter structure has only one chain, chain A, in the asymmetric unit. For the fifth structure, the PSB‐II^142–236^ POG‐PAG‐POG crystal structure, there is also only one chain, chain A, in the asymmetric unit. All PSB‐PSB superpositions were done using the LSQ option of COOT, using the Cα atoms of residues 148–218 for the superposition calculations. The PSB domain and CRTAP (cartilage associated protein, which is a subunit of the collagen prolyl 3‐hydroxylase 1 (P3H1) complex, PDB ID 8K17) were superimposed by LSQ using the Cα atoms of residues 193 to 230 of H9, H10, and H11 of PSB‐I and the corresponding residues (150–187) of CRTAP. Images of the structures were prepared using the PyMOL software [38] (Schrödinger LLC, New York, USA).

## 3 Results and Discussion

### 3.1 The Affinities of Proline‐Rich Peptides for PSB‐I and PSB‐II Are Different

Characterization of the PSB‐I^143–238^ construct by SEC‐MALS shows that it is a monomer in solution (Figure S1), similar to the previously characterized PSB‐II domain [17]. The CD‐melting experiment shows that it is a stable protein, with a *T*_*m*_ of 55.4°C (Figure S2), like the PSB‐II construct (*T*_*m*_ = 60.5°C) [17]. The affinity properties of the PSB‐I and PSB‐II constructs, as obtained from ITC peptide binding experiments, are shown in Table 2 and Figure S3. The PSB‐I construct has a higher affinity for the PxGP‐peptides than for (PPG)_3_ with *K*_*D*_ values of 91.3, 37.0, and 84.3 μM for PPG‐PAG‐PPG, PPG‐PRG‐PPG, and PPG‐PEG‐PPG, respectively, and 309.2 μM for (PPG)_3_ and 80.9 μM for P9 (Figure S3). The affinities of (PPG)_3_ and P9 for the PSB‐I domain of the DD‐I construct have been reported previously as 143.5 and 9.8 μM, respectively [12], showing that the assembly of the PSB‐I domain as an integral part of the DD‐I dimer causes tighter binding of these two peptides. The higher affinity of the PxGP‐peptides compared with the (PPG)_3_ peptide was also observed in the previous studies with PSB‐II [17] (Table 2). The comparison of the affinities of PSB‐I and PSB‐II for these peptides also shows clear differences, in that PSB‐I has a lower affinity than PSB‐II for the PPG‐PAG‐PPG, PPG‐PEG‐PPG, and PPG‐PRG‐PPG peptides, but a higher affinity for the (PPG)_3_ and P9 peptides, and thus the difference between the binding affinities of the PxGP‐peptides when compared with (PPG)_3_ and P9 is much more marked in the case of PSB‐II than PSB‐I. The higher affinity of (PPG)_3_ and P9 for PSB‐I, compared with PSB‐II, is consistent with previously reported affinities of these domains for (PPG)_5_ and poly(L‐Pro) (*M* = 5000) [20] and is also consistent with the classical knowledge that the C‐P4H‐I tetramer can be purified by affinity chromatography using a poly(L‐Pro) column [39], whereas C‐P4H‐II cannot [40]. Furthermore, C‐P4H‐I has lower *K*_*M*_ values for the (PPG)_10_ substrate and markedly lower inhibition constants for poly(L‐Pro) (*M* = 7000) than C‐P4H‐II [40].

**TABLE 2:** ITC binding studies with proline‐rich peptides^a^.

PSB‐I^143–238^ construct^b^					PSB‐II construct^c^
Peptide	*K*_*D*_ (μM)	Δ*H* (kcal/mol)	−*T*Δ*S* (kcal/mol)	Δ*G* (kcal/mol)	*K*_*D*_ (μM)
(PPG)_3_	309.2 ± 9.5	−13.6 ± 1.7	8.77 ± 1.66	−4.83 ± 0.06	Not detectable
P9	80.9 ± 14.8	−9.5 ± 0.8	3.95 ± 0.82	−5.59 ± 0.10	> 1500
PPG‐PAG‐PPG	91.3 ± 3.8	−12.4 ± 0.5	6.86 ± 0.49	−5.51 ± 0.02	19.7 ± 0.7
PPG‐PRG‐PPG	37.0 ± 1.6	−14.9 ± 0.4	8.87 ± 0.37	−6.06 ± 0.04	6.8 ± 1.0
PPG‐PEG‐PPG	84.3 ± 3.0	−13.2 ± 1.2	7.67 ± 1.17	−5.57 ± 0.02	29.2 ± 0.7
POG‐PAG‐POG	79.6 ± 9.6	−12.7 ± 0.2	7.07 ± 0.25	−5.60 ± 0.08	26.2 ± 1.9

^a^ The average values and standard deviations are obtained from three measurements.

^b^ The PSB‐I ITC data were collected and processed as described in Section 2.

^c^ The PSB‐II ITC data have been described previously [17], except for the POG‐PAG‐POG peptide, for which the ITC data were collected and processed as described in Section 2.

### 3.2 The Structures of PSB‐I Complexed With PxGP‐Peptides

**FIGURE 3:**
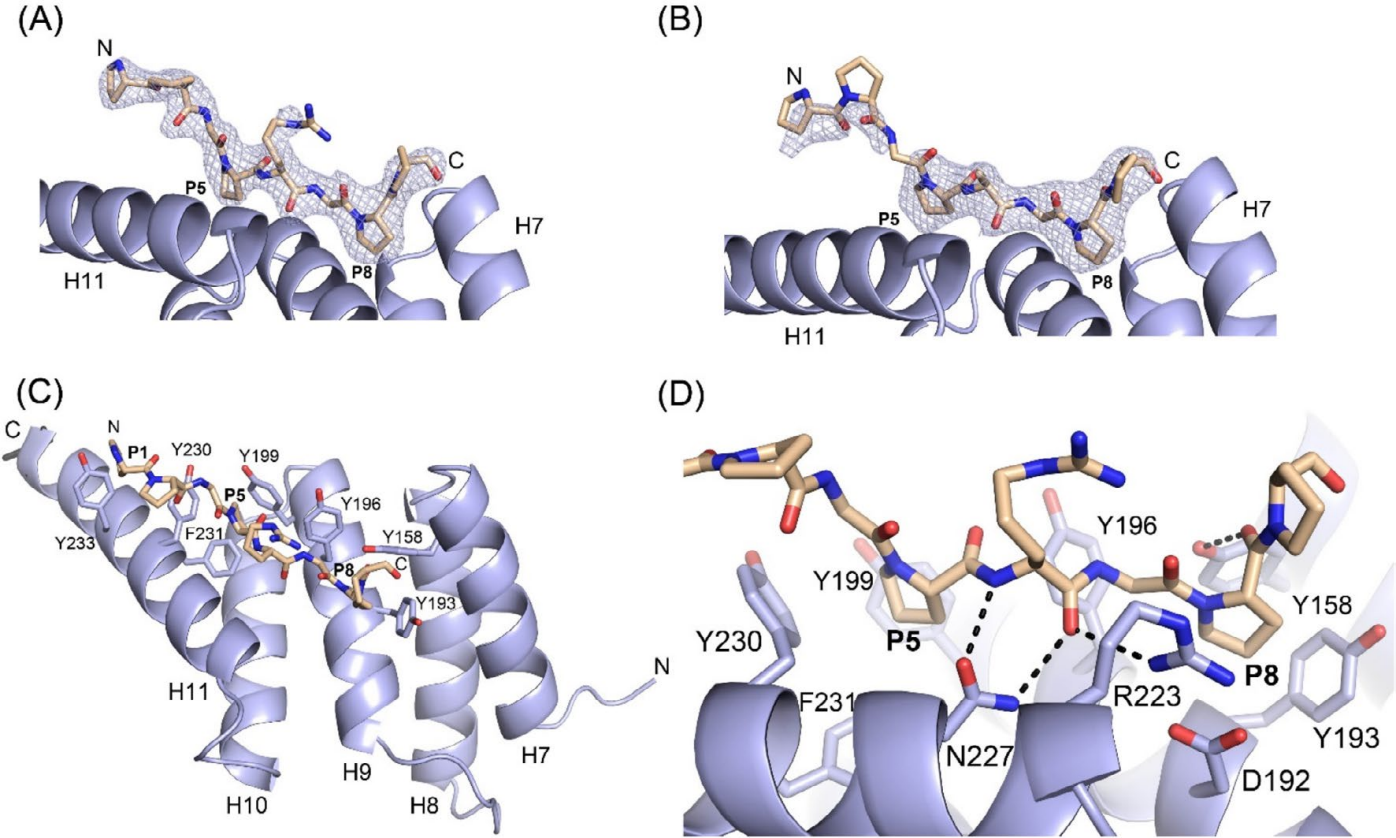
Important structural features of the mode of binding of the PPG‐PRG‐PPG and PPG‐PAG‐PPG peptides to the PSB‐I domain. (A) The (Fo–Fc) omit map of the PSB‐I structure complexed with PPG‐PRG‐PPG (chain B), contoured at three sigma, and calculated from the final model, after refinement with the highlighted peptide omitted. (B) The corresponding (Fo–Fc) omit map of the PSB‐I structure complexed with PPG‐PAG‐PPG (chain B). (C) The proline side chains of residues Pro1, Pro4 and Pro7 of the PPG‐PRG‐PPG peptide point inwards and interact with the side chains of tyrosines in the P1‐, P5‐, and the P8‐pockets, respectively. The P1‐pocket is a shallow pocket, whereas the P5‐pocket and P8‐pocket are deep pockets (a stereo figure is provided as Figure S4). (D) The geometry of the PPG‐PRG‐PPG peptide bound in the P5‐ and P8‐pockets. Dotted lines visualize the four direct hydrogen bonds between the PSB domain and the bound peptide.

The crystal structures of the complexes of the PSB‐I construct with the PPG‐PAG‐PPG peptide (refined at 2.15 Å resolution) and the PPG‐PRG‐PPG peptide (refined at 2.17 Å resolution) (Figure 3) were obtained by cocrystallization (Table 1). The PSB‐I model in both structures extends from Ser143 (H7) to Glu238 (H11), continuing into additional helical residues formed by histidines of the C‐terminal His_6_‐tag. The mode of binding of the peptides in the P5‐ and P8‐pockets is well defined by the electron density map for both structures (Figure 3A,B). In the PPG‐PRG‐PPG crystal form, there are 4 chains per asymmetric unit. In one chain (chain C) no peptide is bound, and in three chains, the peptide is bound, always in the same mode of binding. chain B is used as the reference structure as in this structure, the bound peptide is visible in the electron density map from residues 1 to 8 (the last residue was not built). In the PPG‐PAG‐PPG crystal form, there are two chains in the asymmetric unit, and both chains have a peptide bound in the peptide‐binding groove, adopting the same mode of binding as seen for PPG‐PRG‐PPG. In chain B, the bound peptide could be built from residues 1 to 8, and the structure of the chain B complex is used as the reference structure of the PPG‐PAG‐PPG complex. Figure 3C,D and Figure S4 visualize how the X‐prolines (Pro1, Pro4, and Pro7) of PPG‐PRG‐PPG bind respectively in the P1‐, the P5‐, and the P8‐pockets. Tyr196 contributes to both the P5‐ and the P8‐pocket (Figure 2). The P1‐pocket is rather shallow, whereas the P5‐ and P8‐pockets are deeper pockets.

### 3.3 Structural Differences and Similarities of the Mode of Binding of the PxGP‐Peptides to PSB‐I and PSB‐II

**FIGURE 4:**
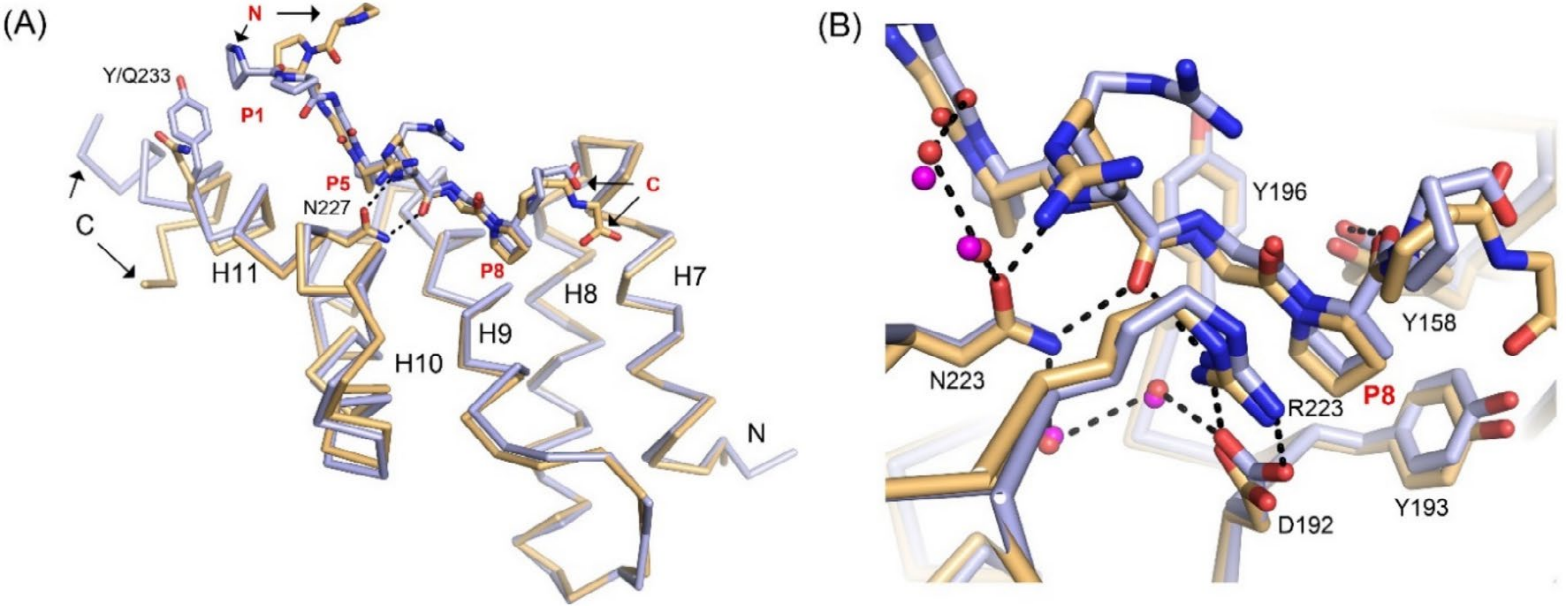
Comparison of the PxGP mode of binding of the PPG‐PRG‐PPG peptide to PSB‐I and PSB‐II (PDB ID 6EVO, chain A). PSB‐I and bound peptide are in cyan color. PSB‐II and bound peptide are in wheat color, respectively. (A) In the PSB‐II complex the N‐terminal end of the peptide does not interact with the PSB domain, as Tyr233 of PSB‐I corresponds to a glutamine in PSB‐II. (B) The P8‐pocket of PSB‐I and PSB‐II is shaped by the side chains of 5 conserved residues: Tyr158, Asp192, Tyr193, Tyr196, Arg223 in PSB‐I. The peptide plane between Arg5 and Gly6 has a stacking interaction with the side chain of Tyr196. Red and magenta dots are water molecules of the PSB‐I and PSB‐II complex, respectively. Dotted lines visualize hydrogen bonds of the PSB‐I complex. A stereo figure is provided as Figure S5.

The mode of binding of these peptides in the P5‐ and P8‐pockets is the same as seen in the structures of the PSB‐II complexes with these peptides [17], but in the latter complexes the N‐terminus of the peptide points into bulk solvent, whereas in both PSB‐I complexes the X‐proline at the N‐terminus of the peptides interacts with the side chain of Tyr233 (Figure 4A; Figure S5). Also, at the C‐terminus of the peptide there are differences in the mode of binding, being better ordered in the PSB‐II complex (Figure 4A; Figure S5). In the PxGP mode of binding, the peptide binds in the peptide‐binding groove, such that the side chain of Asn227 has direct hydrogen bonds with, respectively, the main chain nitrogen and oxygen atoms of Arg5 of the bound peptide (Figures 3D and 4B).

### 3.4 Comparisons of the Structures of the Liganded and Unliganded PSB‐I Chains

**FIGURE 5:**
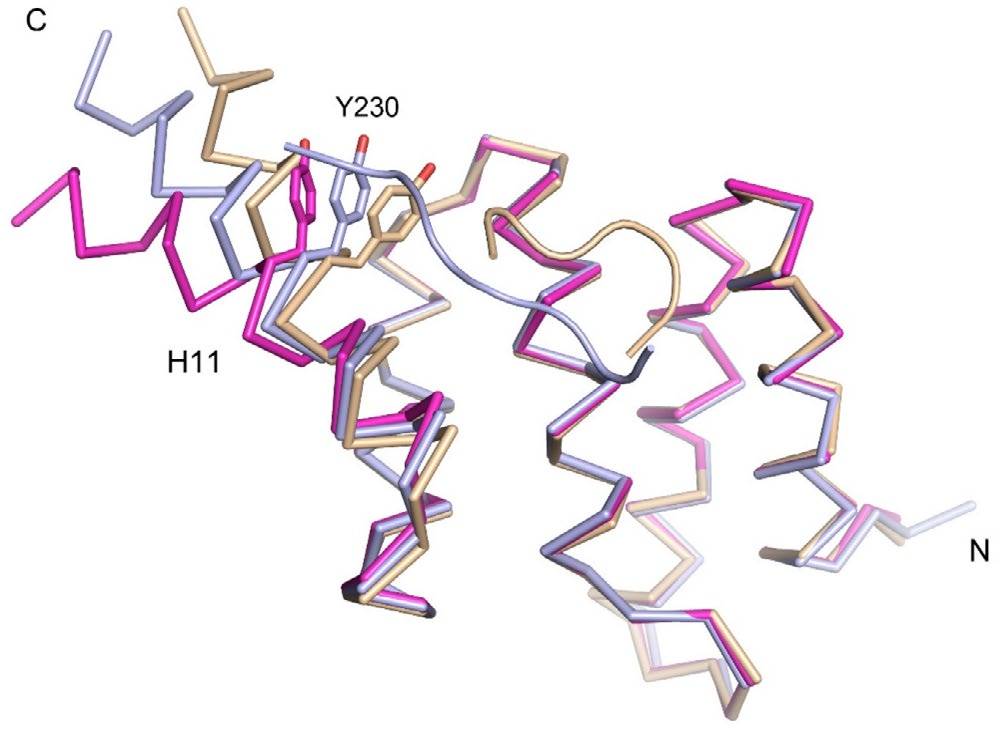
H11 can adopt different conformations. Helix H11 is straight in the structure of liganded chain of PSB‐I^143–238^ (light blue, chain B, complexed with PPG‐PRG‐PPG), but kinked in the unliganded chain (magenta, chain C, H11 is bent away from the core of the domain). H11 is also kinked in the structure of the longer PSB‐I^144–244^ construct (wheat, H11 is bent toward the core of the domain). The bound peptides to chain B of PSB^143–238^ and to chain A of the longer PSB‐I^144–244^ construct are also shown, using the same color code.

The comparison of the structures of the liganded chains (chains A, B and D) and the unliganded chain (chain C) of the PSB‐I PPG‐PRG‐PPG crystal form shows that helix H11 adopts a different conformation in the unliganded chain, pointing more outwards (Figure 5). From residue Tyr230 onwards, helix H11 is bent away from the peptide‐binding groove in the unliganded structure, and the side chain of Tyr230 of the P5‐pocket is partially disordered. In the peptide‐bound complexes (chains A, B and D) the side chain of Tyr230 points to the side chain of Tyr199, without forming a hydrogen bond as the distance between OH(Tyr230) and OH(Tyr199) is 4.1 Å. In the unliganded structure (chain C) the Tyr199 side chain is also partially disordered. It is not clear if this conformational difference of H11 is related to different crystal contacts or due to the absence of the peptide, or both. In any case, the peptide‐binding groove of the unliganded chain C is not blocked by crystal contacts.

### 3.5 Binding Properties of the Conserved P8‐Pocket

**FIGURE 6:**
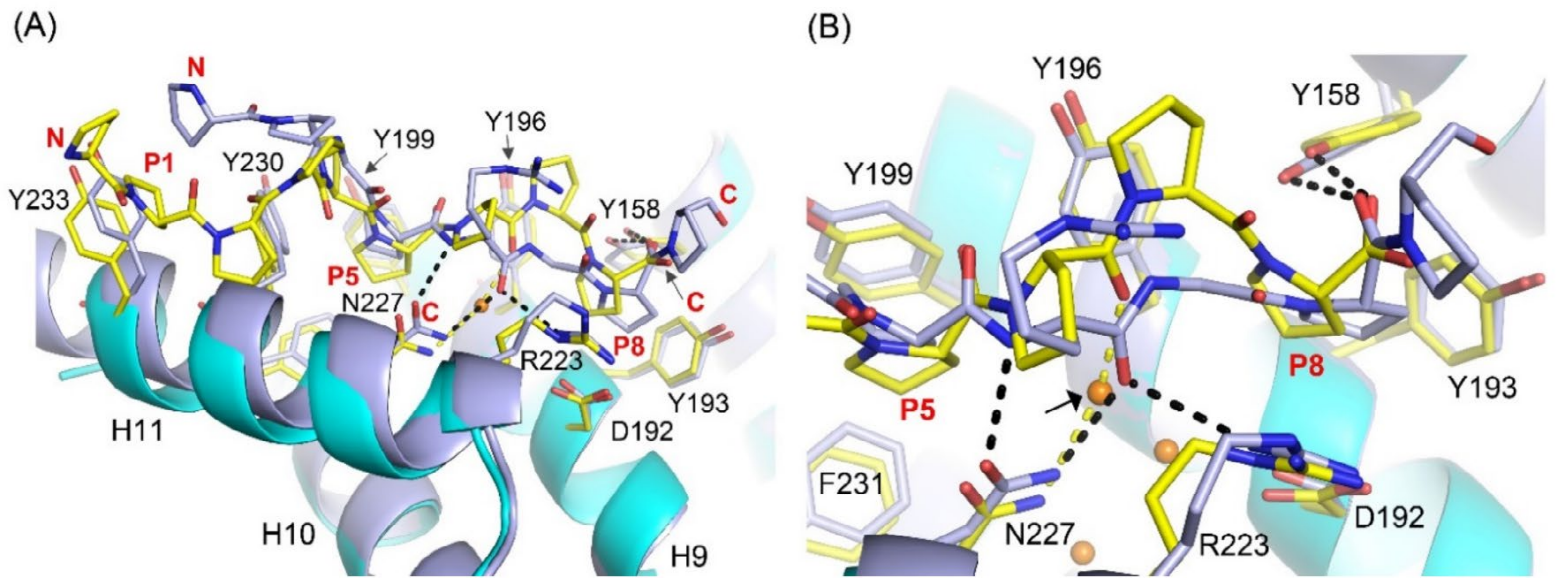
Comparison of the PxGP and PPII mode of binding of proline‐rich peptides to PSB‐I. The PxGP mode of binding of the PPG‐PRG‐PPG complex with PSB‐I (chain B) is in light blue color and the PPII mode of binding of the P9 complex with PSB‐I (PDB ID 4BTB, chain A) is in yellow color. (A) In the PxGP mode of binding the peptide binds more deeply in the peptide‐binding groove between the P5‐ and P8‐pockets, than in the PPII mode of binding (a stereo figure is provided as Figure S8). (B) In the PxGP mode of binding there are four direct hydrogen bonds between the peptide and the PSB domain (visualized by dotted lines), which are with N(Arg5) (by OD1(Asn227)), with O(Arg5) (by ND2(Asn227) and by NH1(Arg223)), and with O(Pro7) (by OH(Tyr158), whereas in the PPII mode of binding there is only one, by OH(Tyr158), interacting with the main chain oxygen atom of residue Pro8 of the P9 peptide. Orange dots are the three water molecules, which form a buried cluster of three water molecules in the PPII mode of binding. The water highlighted with a small arrow is not present in the PxGP mode of binding.

The P8‐pocket is defined by the side chains of five residues (Figures 3D and 4B). The side chains of Tyr158 (H7) and Tyr193 (H9) of the P8‐pocket adopt essentially the same conformation in the liganded (chain B) and unliganded (chain C) structures. The side chain of Tyr158 makes favorable edge‐to‐face van der Waals interactions with the side chain of Tyr193 (Figure S4) [41]. Only the Asp192(H9)–Arg223(H11) salt bridge interaction at the P8‐pocket is less well defined in the unliganded structure, and its geometry is different. In addition to the hydrophobic interactions between the proline ring and the phenyl rings of Tyr158 and Tyr193 in the P8‐pocket, there is also a hydrogen bond interaction between the hydroxyl group of Tyr158 and the main chain oxygen of Pro7 (bound in the P8‐pocket) of the bound peptide. This hydrogen bond interaction is present in both the PPII as well as the PxGP mode of binding with the proline that is bound in the P8‐pocket (Figure 6B). In each of these complexes, the proline side chain pointing into the P8‐pocket is also stacked with the Asp192–Arg223 salt bridge.

In the unliganded structure (chain C) a solute molecule is bound in the P8‐pocket, modeled as a glycine residue. Glycine is a component of the protein buffer. In the unliganded structure of the PSB‐II construct [17] a solute molecule is also bound in the P8‐pocket, also modeled as a glycine. In this respect it is noteworthy to discuss another crystal structure of PSB‐I (of the longer PSB‐I^144–244^ construct), obtained by a crystallization experiment of this PSB‐I construct, without peptide present in the mother liquor. In this structure the C‐terminal His_6_‐tag binds in the nearby peptide‐binding groove of a crystallographically related chain. In this crystal form there is one PSB domain per asymmetric unit and the structure was refined at 2.03 Å resolution (Table 1). The six His_6_‐tag residues are numbered from His(−1) to His4 and the crystal packing is such that one histidine side chain of the His_6_‐tag of a crystallographically related molecule binds in the P8‐pocket (His(−1)) and two other histidine side chains (His2, His3) of the same tag interact, respectively, with Tyr196 and Tyr230 (at the P5‐pocket) (Figures S6 and S7). The best‐defined region of the His_6_‐tag concerns the His2–His3 region (Figure S6). The tyrosine side chains forming the P8‐pocket are in the same conformation, except for the side chain of Tyr196 (between the P5‐ and P8‐pockets). Also, Tyr230 (at the P5‐pocket) adopts a somewhat different side chain conformations in this structure. The P8‐pocket is more rigid than the P5‐pocket (Figure S7). The interactions between the His_6_‐tag peptide and the PSB domain are predominantly stacking interactions between, respectively, histidine side chains of the His_6_‐tag peptide and tyrosine side chains of the PSB‐I domain (Figure S7). It is interesting to note that H11 in this structure is also a kinked helix from residue Tyr230 onwards, pointing inward (opposite direction as described above for the unliganded chain C) (Figure 5). In this structure the Tyr230 side chain has moved toward the side chain of Tyr199, such that there is a hydrogen bond interaction between OH(Tyr230) and OH(Tyr199) (the distance between these two atoms in this structure is 2.4 Å).

### 3.6 The Two Modes of Binding of the Proline‐Rich Peptides

The directionality of the bound PxGP‐peptide is the same as seen for the bound PPII peptide, but the conformation and the details of the mode of binding of the bound peptides are different (Figure 6). In the PPII complexes, the Y‐prolines are bound in the P5‐ and P8‐pockets, whereas the PxGP‐peptides bind with their X‐prolines in the P5‐ and P8‐pockets (Figure 2). The interactions of the proline side chains bound in the P5‐ and P8‐pockets are similar, interacting with the side chains of Tyr199, Tyr230, and Phe231 (in the P5‐pocket) and Tyr158, Tyr193, and the Asp192–Arg223 salt bridge (in the P8‐pocket) (Figure 6B). In addition, in both modes of binding, the conserved Tyr196 interacts with a peptide plane of the bound peptide, being the Arg5–Gly6 peptide moiety of the PxGP‐peptide PPG‐PRG‐PPG (Figure S8), which corresponds to the Gly6‐Pro7 peptide moiety of the PPII mode of binding.

In the PxGP mode of binding, the peptide binds more deeply in the peptide‐binding groove (Figure 6A) as compared with the PPII mode of binding, and there are four direct hydrogen bonds between the PxGP‐peptide and the PSB‐I domain, which are with N(Arg5) (by OD1(Asn227)), with O(Arg5) (by ND2(Asn227) and by NH1(Arg223)), and with O(Pro7) (by OH(Tyr158)), whereas in the PPII mode of binding there is only one, by OH(Tyr158), interacting with the main chain oxygen atom of Pro8 of the PPII peptide (Figure 6B).

A key difference between the PPII (e.g., (PPG)_3_) and PxGP (e.g., PPG‐PRG‐PPG) mode of binding is the two direct hydrogen bonds of the Asn227 side chain with the main chain of the peptide in the PxGP mode of binding, whereas in the PPII mode of binding, the Asn227 side chain hydrogen bond interactions with the peptide main chain are mediated via water molecules. O(Arg5) (of PPG‐PRG‐PPG) replaces a water molecule and consequently, a buried cluster of three waters (in the PPII mode of binding of (PPG)_3_) is changed into a buried cluster of two waters (in the PxGP mode of binding of PPG‐PRG‐PPG) (Figure 6B). Asn227 is fully conserved (Figure 1A), which suggests that this interaction is critically important for the functional role of the PSB domain. Such a functional role is also suggested by the observation that the PxGP‐peptides bind with higher affinity, both to PSB‐I as well as to PSB‐II compared with the (PPG)_3_ peptide (Table 2). In the (PPG)_3_ mode of binding, the peptide adopts the PPII conformation, which is not possible for the PxGP sequence motifbecause the *ϕ*/*ψ* values of residue Arg5 (−105°, 129°) are not possible for a peptide with a proline at this position, as the *ϕ* value for a proline is restricted to approximately *ϕ* = −70^o^ [42], due to the geometry of the proline ring. In the PxGP mode of binding, a residue with a side chain at the glycine position is not allowed as it would clash with Tyr158 and Tyr196.

In the PPII mode of binding, the X‐prolines bind in the P5‐ and P8‐pockets (Figure 2), as observed in the structures of the DD‐I construct complexed with (PPG)_3_ and P9. In the structure of the P9 complex, the X and Y assignments of the peptide cannot be made, as there are no glycines, but using the residue numbering scheme of (PPG)_3_, Pro5 and Pro8 bind in the P5‐ and P8‐pockets, and this region of the peptide is in the same PPII conformation as observed for (PPG)_3_. The crystal packing of the (PPG)_3_ complex and the P9 complex is different, such that the (PPG)_3_ peptide cannot bind in the PPII conformation at its N‐terminal end, whereas the P9 peptide adopts the PPII conformation over its entire length (Figure S8), allowing the proline of Pro1 to stack with the side chain of Tyr233. This Pro1 corresponds to an X‐proline relative to the Y‐prolines Pro5 and Pro8.

### 3.7 The Mode of Binding of the POG‐PAG‐POG Peptide to PSB‐I and PSB‐II

**FIGURE 7:**
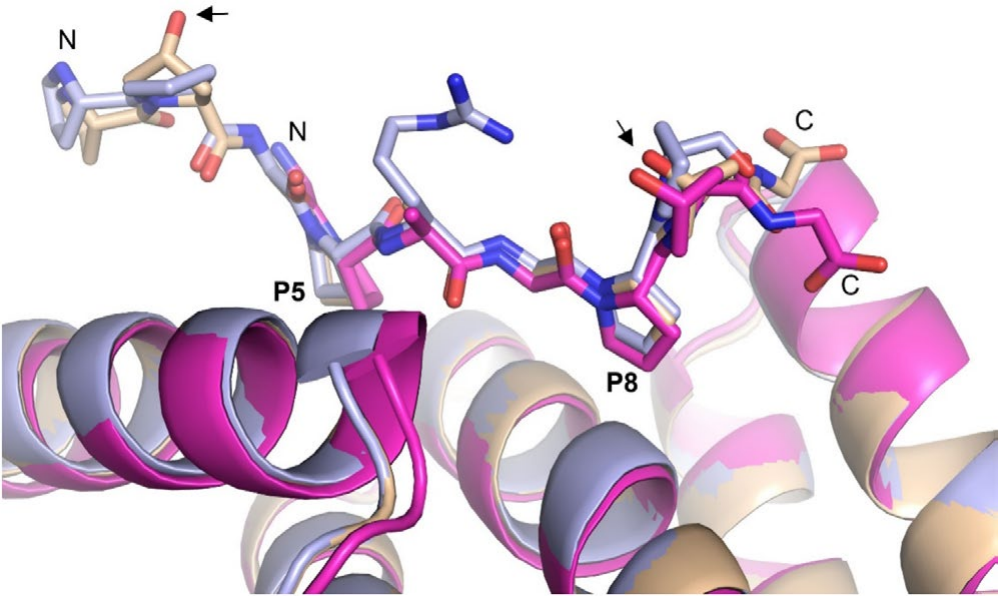
The structures of the complexes of PSB‐I and PSB‐II with the bound POG‐PAG‐POG peptide. The superposition of the structures of the PSB‐I POG‐PAG‐POG complex (wheat), the PSB‐II POG‐PAG‐POG complex (magenta) and the PSB‐I PPG‐PRG‐PPG complex (cyan) visualizes the PxGP mode of binding of the POG‐PAG‐POG peptide in these complexes. The 4‐hydroxy groups of the hydroxyprolines (identified by small arrows) point toward bulk solvent.

Prolines that occur in the PxGP motif cannot be hydroxylated by the C‐P4H catalytic domain, as they are in the X‐position of the XYG‐repeat and the x‐residue at the Y‐position cannot be a proline. Previous C‐P4H peptide binding studies have shown that the (Gly‐Pro‐4Hyp)_5_ model peptide, which has a hydroxyproline at the Y‐position of each tripeptide repeat, has much lower affinity than the (PPG)_5_ substrate peptide for the PSB‐I and PSB‐II constructs [20] and the (Gly‐Pro‐4Hyp)_5_ peptide is not a substrate of C‐P4H‐I or C‐P4H‐II [20]. Low affinity of the (Gly‐Pro‐4Hyp)_5_ peptide for the PSB construct is consistent with the notion that in the PPII mode of binding of the (PPG)_3_ peptide the Y‐prolines Pro5 and Pro8 are pointing into the P5‐ and P8‐pockets (Figure 2) and the presence of the hydroxyl groups of the hydroxylated peptide would cause clashes with the protein part of the P5‐ and P8‐pockets. However, in the PxGP mode of binding, the side chains of the Y‐prolines Pro2 and Pro8 point into solution, suggesting that such hydroxylated peptides could have affinity for the PSB domain. In order to test this hypothesis, the binding properties of the POG‐PAG‐POG peptide for PSB‐I and PSB‐II were also studied. In this peptide the Pro2 and Pro8 Y‐prolines are hydroxylated (as the 4‐hydroxy moiety), and therefore this peptide is also a homolog of a partially hydroxylated collagen chain. Calorimetric binding data show that this peptide binds with good affinity to the PSB‐I and PSB‐II constructs, having, respectively, a *K*_*D*_ of 79.6 and 26.2 μM, which is very similar to that observed for the non‐hydroxylated PxGP‐peptides (Table 2). Crystal structures of PSB‐I and PSB‐II complexed with this peptide were also obtained. In the crystallization experiments with the PSB‐I construct, the same crystal form was obtained as for the PPG‐PRG‐PPG complex. The structure was refined at 2.05 Å resolution (Table 1) and Chain B is again used as the reference structure. In this chain the bound peptide has been built for residues 1 to 9 (Figure S6B). The peptide shows a very similar mode of binding as the PPG‐PAG‐PPG and PPG‐PRG‐PPG peptides at the N‐terminus and in the P5‐ and P8‐pockets (Figure 7). The protein crystallographic binding studies of the PSB‐II construct with the POG‐PAG‐POG peptide provided a 1.75 Å resolution structure of this complex. There is only one chain in the asymmetric unit. The mode of binding of the peptide is well defined for residues 3 to 9 (Figures S6C and S6D). The 4‐hydroxy groups of the hydroxyprolines of Pro2 (in the PSB‐I complex) and Pro8 (in the PSB‐I and PSB‐II complexes) point into solution (Figure 7).

### 3.8 Unique Properties of the PSB‐I Domain

The available affinity data show that PSB‐I has markedly better affinity for (PPG)_3_ and P9 than PSB‐II (Table 2). In the P9 PSB‐I complex, the side chain of a proline at the N‐terminus of the peptide interacts tightly with Tyr233 (Figure 6; Figure S8), which is not seen in the structure of the (PPG)_3_ complex due to interactions of the latter peptide with a crystallographically related PSB domain. The structural data therefore show that Tyr233 interacts with a proline if the bound proline‐rich peptide adopts the PPII conformation over its entire length [12]. These PSB‐peptide interactions with Tyr233 exist therefore for the PPII mode of binding [12] and for the PxGP mode of binding (Figures 2 and 3) and they include interactions with Tyr230, which, together with Tyr233, forms the shallow P1‐pocket (Figures S4 and S8). Mutagenesis data also confirm that Tyr233 is important for high affinity binding of (PPG)_10_ and poly(L‐Pro) to the PSB‐I construct [15] as well as to the C‐P4H‐I tetramer [14, 15]. The structural (Figure 6A) and ITC data (Table 2), as well as the sequence conservation of Tyr233 in PSB‐I (Figure 1A) suggest that Tyr233 provides unique functional properties to the PSB‐I domain. Apparently, C‐P4H‐I has therefore more recognition sites for binding to collagen polypeptides as compared with C‐P4H‐II, suggesting that C‐P4H‐I is more important for the hydroxylation of collagen. This correlates with the observation that the *P4ha1* knockout mice (lacking C‐P4H‐I) are not viable, whereas *P4ha2* knockout mice (lacking C‐P4H‐II) are viable and do not manifest any major phenotypic abnormalities [3, 43, 44].

### 3.9 The PSB Domain and Its Position in the C‐P4H Tetramer

The crystal structure of the DD‐I construct [12] shows how the PSB domain interacts with the N‐terminal dimerization domain. The dimerization domain consists of two long helical regions (H1, H3, and H4) which interact with the same regions of the second α‐subunit of the α_2_‐dimer, forming a coiled‐coil dimerization motif (Figure 1C). The region of the N‐terminal domain which follows the coiled‐coil region consists of two helices, H5 and H6, which interact with the H7‐H8 helices of the PSB domain (Figure 1B). Topologically, helices H5 and H6 extend the TPR fold [16] of the PSB domain at its N‐terminus by one repeat. The C‐terminal region of the PSB domain, H11, continues into the linker region connecting the PSB domain and the CAT domain, which starts with helix H12 [9]. In the model of the tetramer (Figure 1C), there are no direct interactions between the PSB domain and the CAT domain. However, structural changes of the C‐terminal end of H11, as observed from the comparison of the various PSB structures (Figure 5), will affect the structure of the linker region between the PSB and CAT domains (Figure 1C) and therefore could affect the orientation and position of the PSB domain with respect to the CAT domain. Conformational changes indeed were detected in SAX studies of C‐P4H‐I when adding peptides such as (PPG)_8_ and (Pro)_24_ [8]. In any case, the peptide bound to the PSB groove points with its C‐terminus to the catalytic domain (and therefore to the N‐terminus of the peptide bound at its active site) [9], which is consistent with the observation that the C‐terminal prolines of the 9th and 4th triplet of the (PPG)_10_ and (PPG)_5_ model peptides, respectively, are preferentially hydroxylated by C‐P4H [45, 46].

**FIGURE 8:**
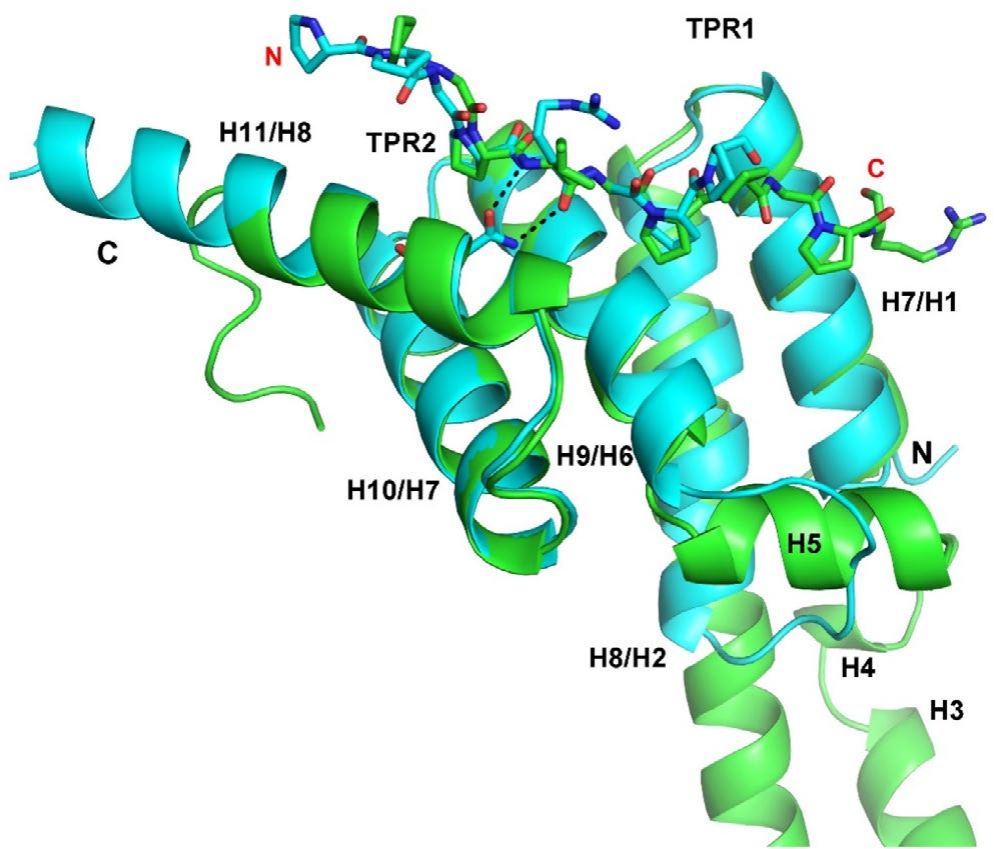
Superposition of the TPR units of the PSB domain of C‐P4H‐I (cyan) (complexed with PPG‐PRG‐PPG) and of the CRTAP subunit of the P3H1 complex (green) (PDP ID 8K17). The PxGP proline‐rich peptides are bound in the same conformation to the peptide‐binding grooves of these two domains. The two direct hydrogen bonds between the bound peptide and the side chain of Asn227 of PSB‐I, which corresponds to Asn184 of the CRTAP subunit of the P3H1 complex, are shown as dotted lines. The helices of the PSB domain are labeled as H7/H8 (TPR1), H9/H10 (TPR2) and H11 (solvating helix) and the names of the corresponding helices of CRTAP (H1/H2, H6/H7 and H8) are also provided. In the CRTAP subunit there is an insertion after H2 consisting of three helices, H3, H4, and H5. The x‐residue of the PxGP‐peptide is an arginine in the PSB‐I complex and an isoleucine in the CRTAP complex. The sequence of the modeled peptide bound to PSB‐I is PPG‐PRG‐PP (cyan) and the sequence of the modeled peptide bound to CRTAP is PG‐PIG‐PPG‐PR (green).

Enzyme kinetic studies of C‐P4H, that were done with full‐length procollagen polypeptides, suggest that the enzyme has processivity properties [47]. Such substrate processivity could in principle be explained by a sliding mechanism [48], in which a region of the procollagen chain is captured first by the PSB domain and subsequently this PSB‐bound (XYG)_3_ region slides to the catalytic site of the CAT domain such that it hydroxylates the Y‐proline of the middle repeat of the previously captured (XYG)_3_ region. However, the large distance between the PSB peptide‐binding groove and the CAT peptide–binding groove (Figure 1C) does not favor such a simple sliding mechanism. It is also discussed by Prockop and coworkers [45] that the hydroxylation pattern of the (PPG)_10_ model substrate is not consistent with a simple sliding processivity mechanism. These observations suggest therefore another role of the PSB domain in the C‐P4H reaction mechanism. For example, like a “hold‐and‐bite” mechanism, as described for the DegP protease where there is also a large distance between the anchoring site of the polypeptide substrate and the protease catalytic site [49]. In this respect, it can be noted that recent cryoEM studies of human collagen prolyl 3‐hydroxylase 1 (P3H1) suggest that also in this enzyme the procollagen binding site is not immediately adjacent to the catalytic site [50]. In this structure, consisting of an assembly of three proteins (referred to as the PCP complex), being P3H1, CRTAP, and PPIB, a procollagen binding site located on CRTAP is observed, not near the catalytic site of P3H1. This binding site is also formed by two TPR units plus a solvating helix and the collagenous peptide is bound in the PxGP conformation (Figure 8). The P5‐ and P8‐pockets of the CRTAP peptide‐binding groove are both shaped by conserved tyrosines and phenylalanines. The interactions of the two proline side chains of the PxGP motif and the direct hydrogen bonds between the main chain of the x‐residue of the bound peptide and the side chain of the conserved asparagine of CRTAP are also preserved (Figure 8).

## 4 Concluding Remarks

The PSB domain is a functionally important domain of C‐P4Hs, as for example, mutagenesis studies show that its binding properties are needed for efficient hydroxylation of (PPG)_10_ [15]. Here it is shown that collagenous, proline‐rich peptides can bind in two possible conformations to the PSB‐I domain, being the extended PPII conformation, such as (PPG)_3_, and the more deeply bound PxGP conformation (Figure 6). In both conformations, proline side chains bind in the P5‐ and P8‐pockets. The high sequence conservation of Asn227, as well as (ii) the direct hydrogen bond interactions of this side chain with the peptide NH and peptide carbonyl oxygen of residue x at position 5 of the PPG‐PAG‐PPG, PPG‐PRG‐PPG, and POG‐PAG‐POG peptides and (iii) the high affinity of these peptides for the PSB domains (Table 2) suggest that the PxGP mode of binding is functionally relevant. This is also in line with the notion that this mode of peptide‐protein complex formation is also seen in the structure of the human P3H1 complex (Figure 8). In the PxGP mode of binding, the x‐residue cannot be a proline; therefore, this mode of binding of the procollagen polypeptide to the PSB domain is not consistent with a simple sliding mechanism, in which the to‐be‐hydroxylated triplet first binds to the PSB domain and subsequently is transferred directly to the CAT domain to become hydroxylated. In the PPII conformation, the Y‐prolines bind in the P5‐ and P8‐pockets, whereas in the PxGP mode of binding, X‐prolines bind in the P5‐ and P8‐pockets (Figure 2). Binding studies with the POG‐PAG‐POG peptide (being hydroxylated at Y‐prolines of the first and the third XYG repeat) have shown that in the PxGP mode of binding, these Y‐prolines can be 4‐hydroxyprolines, indicating that C‐P4H‐I and C‐P4H‐II can bind to unhydroxylated procollagen as well as (partially) hydroxylated collagen chains. Further structural enzymological C‐P4H studies using longer peptides with sequences that are identical to extended fragments of procollagen polypeptides have been initiated to obtain better understanding of the function of the PSB domain.

## Author Contributions

**M. Mubinur Rahman:** investigation, methodology, formal analysis. **Ramita Sulu:** investigation, methodology. **Bukunmi Adediran:** investigation, methodology. **Hongmin Tu:** methodology, writing – review and editing, validation. **Antti M. Salo:** methodology, writing – review and editing, validation. **Sudarshan Murthy:** methodology, writing – review and editing. **Johanna Myllyharju:** writing – review and editing, funding acquisition, project administration, supervision, resources. **Rik K. Wierenga:** funding acquisition, writing – original draft, conceptualization, writing – review and editing, project administration, supervision, resources. **M. Kristian Koski:** conceptualization, funding acquisition, writing – original draft, visualization, writing – review and editing, project administration, supervision, resources.

## Conflicts of Interest

The authors declare no conflicts of interest.

## Supporting information


**Data S1.** prot26839‐sup‐0001‐Figures.

## Data Availability

The crystal structures and their structure factors have been deposited in the PDB. The PDB IDs are provided in Table 1. All other data involved in this work are available from the authors upon request.
