# Supplementary material for: Binding Differences of the Peptide‐Substrate–Binding Domain of Collagen Prolyl 4‐Hydroxylases I and II for Proline‐ and Hydroxyproline‐Rich Peptides
Source: Proteins. 2025 May 19;93(10):1732–46. doi: 10.1002/prot.26839 (PMC12433258; doi:10.1002/prot.26839)
Supplement: Supplementary file 1 — Data S1. prot26839‐sup‐0001‐Figures. [file PROT-93-1732-s001.pdf]

SUPPORTING INFORMATION

Binding differences of the peptide-substrate-binding domain of collagen prolyl 4-hydroxylases I and II for proline- and hydroxyproline-rich peptides

M. Mubinur Rahman[1,*], Ramita Sulu[1,*], Bukunmi Adediran[1,¶], Hongmin Tu[2], Antti M. Salo[1], Sudarshan Murthy[1], Johanna Myllyharju[1], Rik K. Wierenga[1,#], M. Kristian Koski[1,2,#]

[1] Faculty of Biochemistry and Molecular Medicine, University of Oulu, P.O. Box 5400, FI-90014 University of Oulu, Finland
[2] Biocenter Oulu, University of Oulu, P.O. Box 5000, FI-90014 University of Oulu, Finland

[*]Joint first authors
[#]Joint corresponding authors

[¶]Current address: EMBL Grenoble, 71 Avenue des Martyrs, CS 90181, 38042 Grenoble Cedex 9 France

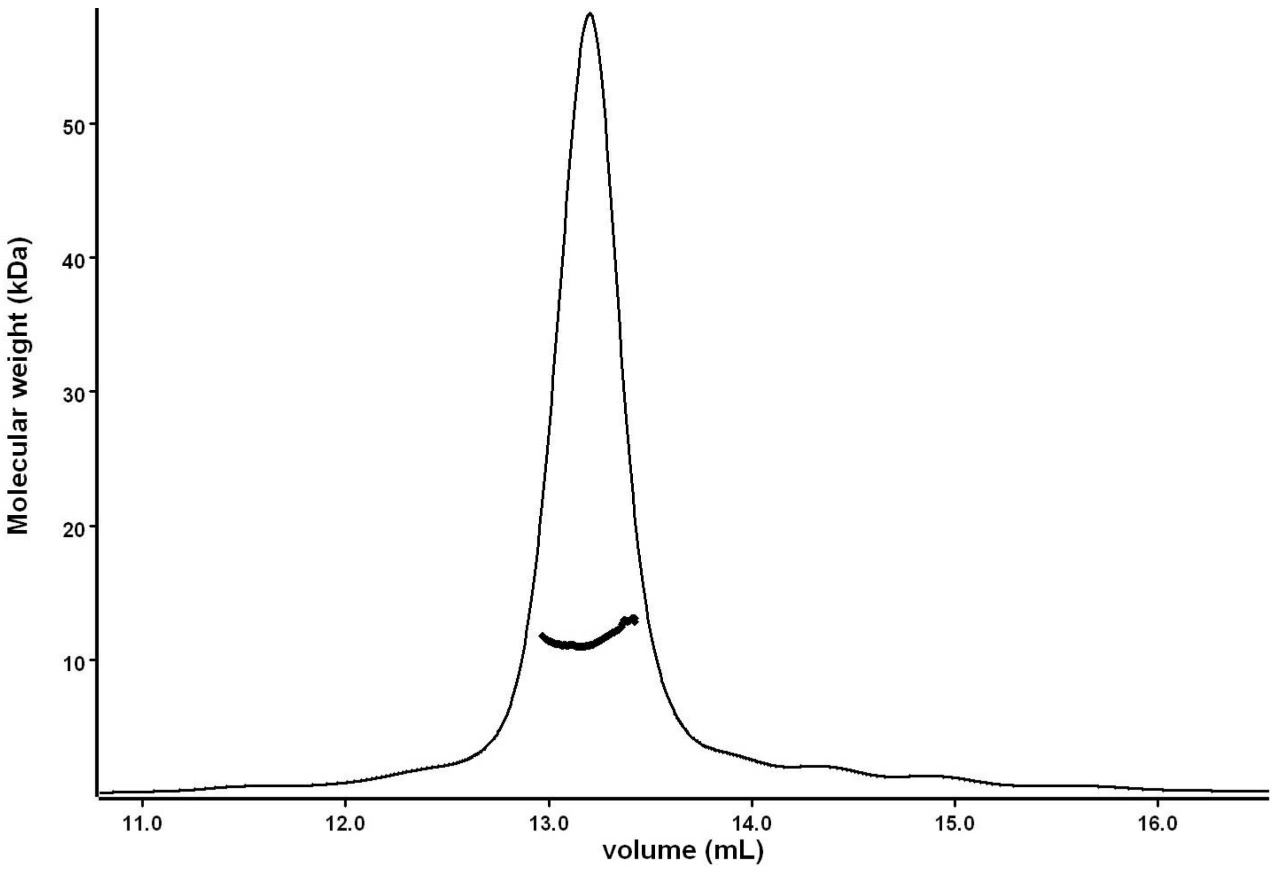

FIGURE S1 The SEC-MALS analysis of the PSB-I$^{143\text{-}238}$ construct. The plot of molecular weight (bold line) *versus* elution time is shown. Also shown is the corresponding light scattering signal (thin line, measured at 90°) showing the PSB-I peak. The calculated molecular weight of 11.5 kDa is close to the theoretical monomeric molecular weight of the PSB-I$^{143\text{-}238}$ construct being 12.4 kDa.

(A)

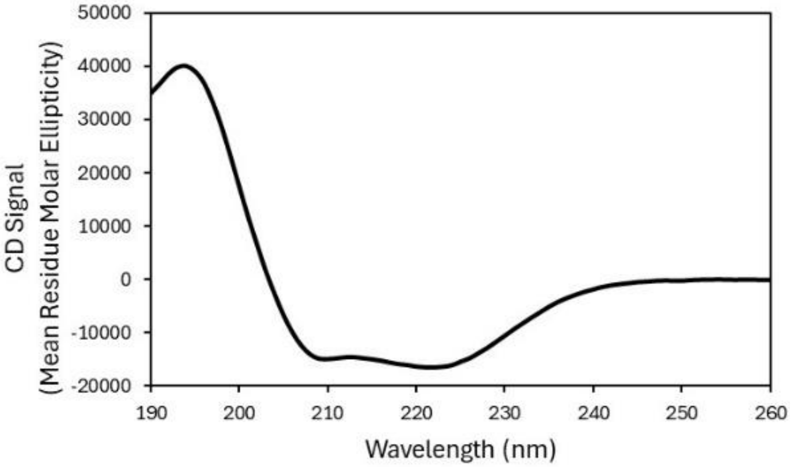

(B)

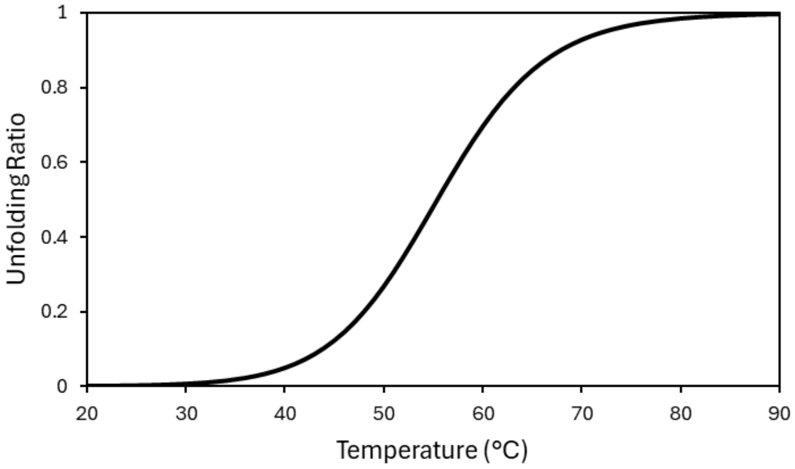

FIGURE S2 CD spectroscopy analysis of PSB-I$^{143\text{-}238}$. (A) Far UV CD spectrum. (B) CD melting curve: the unfolded protein fraction is plotted as a function of temperature, with a calculated T_m of 55.4 ˚C.

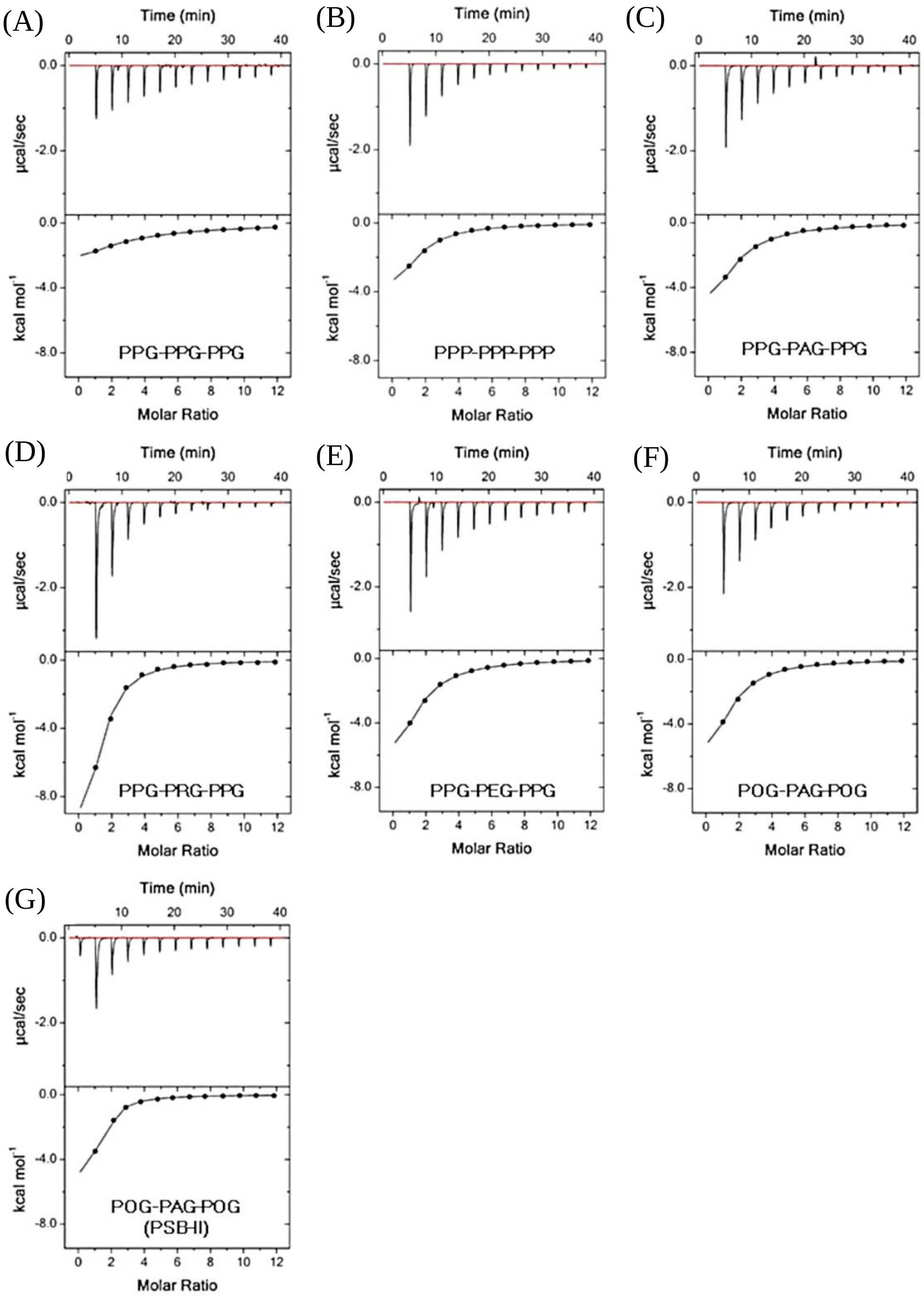

FIGURE S3 Calorimetric binding studies of PSB-I$^{143-238}$ and PSB-II with proline-rich peptides. The titration curves with subtracted baselines (red) of PSB-I with (A) $(PPG)_3$, (B) P9, (C) PPG-PAG-PPG, (D) PPG-PRG-PPG, (E) PPG-PEG-PPG and (F) POG-PAG-POG as well as of (G) PSB-II with POG-PAG-POG are shown. The bottom graphs of each panel display normalized integration data (black spheres) and fitted curves (black lines).

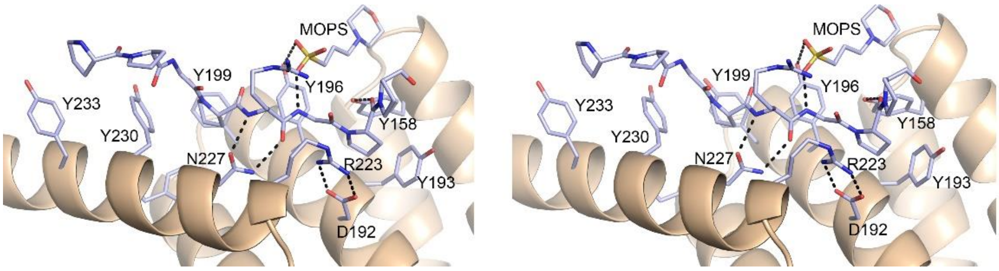

FIGURE S4 Stereo figure showing the mode of binding of the PPG-PRG-PPG peptide in the peptide-binding groove of PSB-I. Pro1 of the peptide interacts with Tyr233 in the rather shallow P1-pocket. Pro4 and Pro7 point inwards, into the P5- and P8-pockets, being stacked in particular with the side chains of Tyr199, Tyr230 and Tyr158, respectively. Also shown is the MOPS molecule. Its mode of binding is stabilized by crystal packing contacts at each of the chains of the PPG-PRG-PPG, PPG-PAG-PPG and POG-PAG-POG PSB-I complexes. The MOPS binding site is near the peptide-binding groove and it is hydrogen bonded with its sulfonate group to the bound peptide, but it is also bound to the unliganded chain C of the PPG-PRG-PPG and POG-PAG-POG PSB-I complex structures.

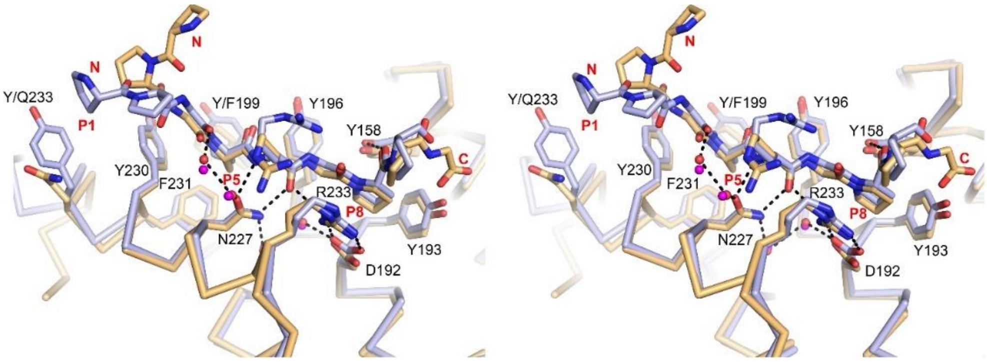

FIGURE S5 This stereo figure compares the mode of binding of the PPG-PRG-PPG peptide to the PSB-I and PSB-II domains, showing the similarities of the PSB interactions in the P5- and P8-pockets of PSB-I (cyan) and PSB-II (wheat) (PDB ID 6EVO). The different interactions at the N-terminal regions of the peptides are also shown: the peptide interacts with its N-terminal proline with the side chain of Tyr233 (Y233) only in PSB-I. The latter residue is a glutamine in PSB-II (**Figure 1A**). At the C-terminus the peptide bound to the PSB-II domain is better ordered. Dotted lines visualize hydrogen bonds.

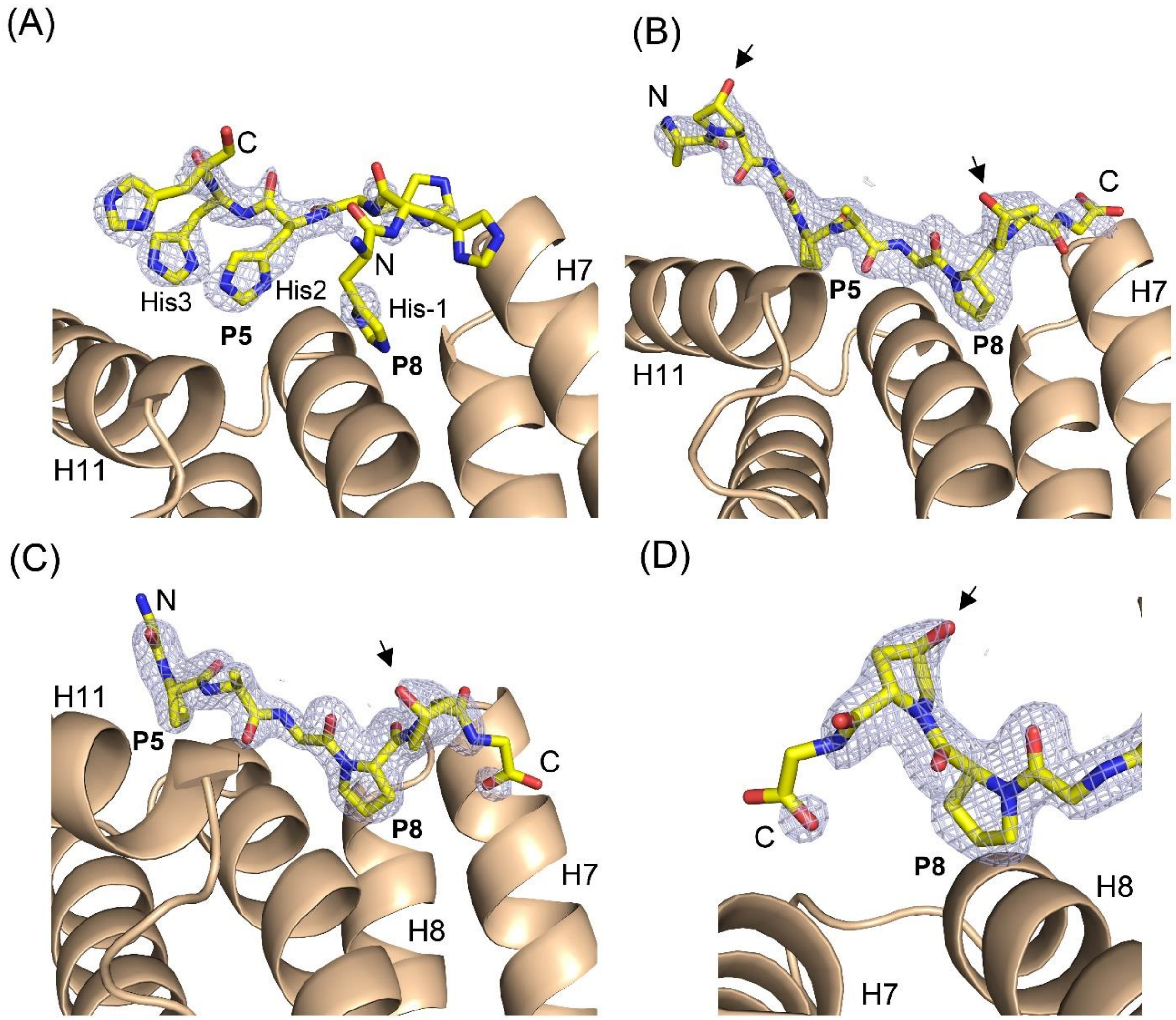

FIGURE S6 The (Fo-Fc) omit maps, contoured at 3 sigma, calculated from the final models, after refinement with the highlighted peptide omitted. (A) PSB-I complexed with the C-terminal His_6-tag of a crystallographically related molecule. (B) PSB-I complexed with POG-PAG-POG (chain B). (C) PSB-II complexed with POG-PAG-POG. (D) PSB-II complexed with POG-PAG-POG (rotated view to highlight the electron density near the 4-hydroxy moiety of the hydroxyproline). The 4-hydroxy moieties of the hydroxyprolines are highlighted by a small arrow in panels (B), (C) and (D). N and C label the N- and C-terminus of the bound peptide. P5 and P8 identify the P5- and P8-pockets of the PSB domain.

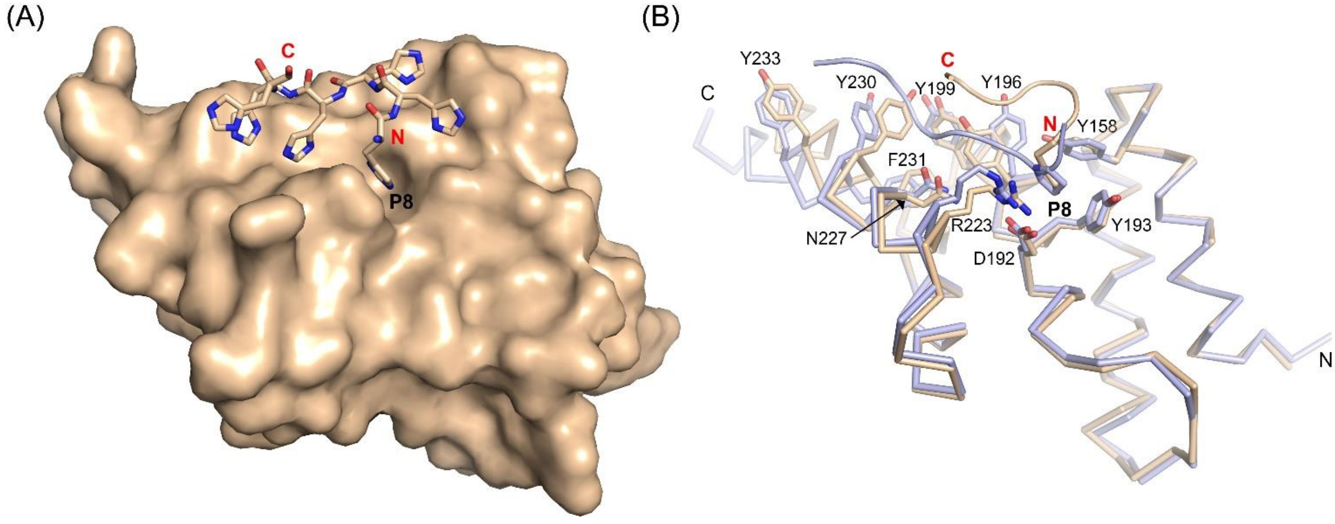

FIGURE S7 Mode of binding of the His_6-tag to the PSB-I domain. The labels N and C identify the N-terminus and C-terminus of the His_6-tag. (A). The side chain of His(-1) binds in the P8-pocket. (B) Superposition of the PSB-I domain complexed with the His_6-tag (wheat) and the PSB-I domain complexed with the PPG-PRG-PPG peptide (cyan). The histidine side chain of the His_6-tag, His(-1), which binds in the P8-pocket, overlaps precisely with the proline side chain of Pro7 of the PPG-PRG-PPG peptide. The side chains of Tyr230 and Tyr196 have adopted different conformations, but the shape of the P8-pocket is well preserved in both structures.

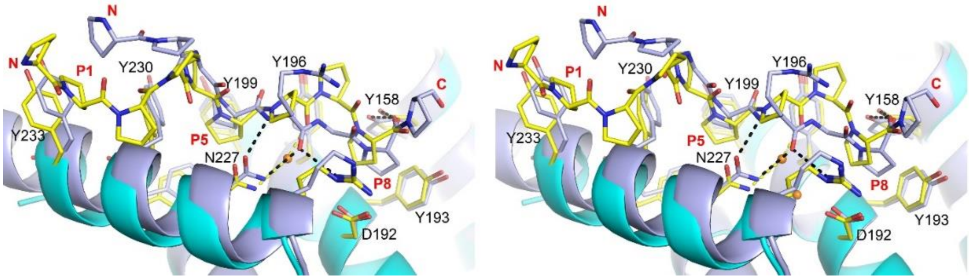

Figure S8 Comparison of the mode of binding of the peptide to the PSB-I domain of the PPG-PRG-PPG peptide (cyan, PxGP conformation) and the P9 peptide (yellow, PPII conformation) (PDB ID 4BTB) (stereo figure). In the P9 complex the peptide adopts the PPII conformation and in the PPG-PRG-PPG complex the peptide adopts the PxGP conformation. In the P5-pocket the proline ring has stacking interactions with the Tyr199 and Tyr230 side chains and in the P8-pocket with the Tyr158 side chain. The orange dots are water molecules of the buried water cluster of the PSB-I complex with bound P9.
